# Therapeutic Delivery of circDYM by Perillyl Alcohol Nanoemulsion Alleviates LPS‐Induced Depressive‐Like Behaviors

**DOI:** 10.1002/advs.202414559

**Published:** 2025-03-27

**Authors:** Feng Gao, Zhongkun Zhang, Minzi Ju, Liang Bian, Huijuan Wang, Sibo Zhao, Ningbo Cai, Yu Wang, Yanpeng Jia, Ling Shen, Yuan Zhang, Honghong Yao

**Affiliations:** ^1^ Department of Pharmacology Jiangsu Provincial Key Laboratory of Critical Care Medicine School of Medicine Southeast University Nanjing Jiangsu 210009 China; ^2^ Co‐innovation Center of Neuroregeneration Nantong University Nantong Jiangsu 226001 China; ^3^ Institute of Life Sciences Key Laboratory of Developmental Genes and Human Disease Southeast University Nanjing Jiangsu 210096 China

**Keywords:** circRNA, depression, lipid nanoparticle, nanoemulsion

## Abstract

RNA therapeutics have recently shown significant promise in treating a variety of diseases, including cancers, infectious diseases, and genetic disorders. However, their potential in addressing neuropsychiatric disorders has been limited by the lack of an efficient delivery system to the central nervous system (CNS). Here, a novel nanoemulsion platform, perillyl alcohol nanoemulsions (PANEs) is introduced, designed for the therapeutic delivery of circDYM to brain and alleviated depressive‐like behaviors. PANEs successfully encapsulated circDYM using cationic lipid compositions, enhancing its delivery efficiency to the mouse brain via its perillyl alcohol phase. The optimized formulation, PANE2‐4, significantly improved the brain delivery efficiency of circDYM compared to both free circDYM and standard lipid nanoparticle formulations. Intranasal administration of PANE2‐4‐circDYM effectively alleviated depressive‐like behaviors in the LPS‐induced mouse model of depression. This effect is achieved by reducing the CD11b^+^CD45^dim^ microglia population and iNOS expression, restoring the expression of protein‐95 (PSD‐95) and synaptophysin. These findings indicated that PANE represents an efficient platform for delivering circRNAs to the brain, and intranasal administration of PANE2‐4‐circDYM is a promising strategy for ameliorating LPS‐induced depressive‐like behaviors.

## Graphical abstract

PANEs are specifically designed to enhance the brain delivery efficiency of circDYM compared to commercialized lipid nanoparticles (LNPs). Intranasal administration of PANE‐circDYM effectively alleviated LPS‐induced depressive‐like behaviors by mitigating neuroinflammation and promoting neuroplasticity. The histological assessment showed PANEs as a safe and effective RNA delivery system, highlighting the therapeutic potential of PANE2‐4‐circDYM for major depressive disorder.

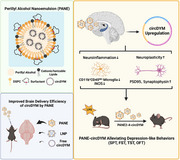

## 1 Introduction

Major depressive disorder (MDD) is a neuropsychiatric disorder characterized by a lack of pleasure and behavioral despair, significantly impacting mood, cognition, behavior, and physical health. Since the onset of the COVID‐19 pandemic, over 50 million cases of MDD have been identified globally.^[^ ^1^, ^2^ ^]^ Despite advances in treatment, the clinical outcomes of current antidepressant drugs remain suboptimal, with only ≈30% of patients achieving remission after first‐line treatment, leaving ≈70% with inadequate responses.^[^ ^3^ ^]^ This limited efficacy is partly attributed to poor access of these drugs to the brain microenvironment, hindering their ability to effectively restore neuronal and glial cell function. Consequently, there is a critical need for novel antidepressant therapeutics that can more effectively target the brain, thereby addressing these shortcomings and improving therapeutic outcomes.

In vitro‐transcribed (IVT) circular RNA (circRNA) has recently emerged as a novel RNA modality with therapeutic potential for treating various diseases, including cancers and infectious diseases.^[^ ^4^, ^5^ ^]^ However, the therapeutic potential of IVT circRNA, particularly in neuropsychiatric disorders, remains largely unexplored. Recent transcriptome analyses have identified multiple circRNAs with aberrant expression in the peripheral blood of MDD patients and animal models of depression, highlighting their potential as therapeutic targets.^[^ ^6^, ^7^ ^]^ Our previous studies identified endogenous circDYM as a key regulator in MDD, showing that it mechanistically binds to TATA‐box binding protein associated factor 1 (TAF1) or miRNA‐9, thereby inhibiting microglia activation.^[^ ^8^ ^]^ Utilizing lentivirus (LV)‐ or extracellular vesicle (EV)‐based technologies, we demonstrated that upregulation of circDYM ameliorated depressive‐like behaviors in both lipopolysaccharides (LPS)‐induced and chronic unpredictable stress (CUS)‐induced depression mouse models.^[^ ^8^, ^9^ ^]^ Despite these promising findings, the use of LVs or EVs for circDYM upregulation is limited by biological toxicities and heterogeneity of vehicle structures.^[^ ^10^, ^11^ ^]^ Given these limitations, we envisioned IVT circDYM with low immunogenicity and high stability as a promising and alternative therapeutic modality, which not only overcomes the drawbacks associated with viral and EV‐based delivery systems but also enhances brain delivery, making it an effective strategy for the treatment of MDD.^[^ ^12^ ^]^

Similar to synthetic linear mRNA or oligonucleotides, IVT circRNA can be encapsulated and delivered via lipid nanoparticles (LNPs) through electrostatic interactions between nucleic acids and ionizable lipids. Although current LNP compositions are often considered safe and stable as drug delivery systems for clinical translation, they are mainly used for systemic administration with high liver clearance due to their ability to form protein corona. Consequently, without additional modifications, very few IVT circRNAs reach the brain microenvironment when delivered by these LNPs.^[^ ^13^ ^]^ In recent years, various strategies have been developed to facilitate brain‐targeted delivery of RNA therapeutics. For instance, Johnsen et al. conjugated anti‐CD71 antibodies (OX26) on the LNPs to improve drug delivery across the blood‐brain barrier (BBB).^[^ ^14^ ^]^ However, the maleimide reaction during OX26 conjugation is reversible and unsuitable for clinical translation. Alternatively, Ma et al. synthesized neurotransmitter‐derived lipidoids for intravenous delivery of amphotericin B and antisense oligonucleotides to the brain.^[^ ^15^ ^]^ Nonetheless, the toxicity and stability of these lipidoids require further investigation. Other studies have demonstrated that local injection, such as intracranial or intracerebroventricular administration, can successfully deliver unmodified LNPs containing RNA therapeutics to the brain microenvironment.^[^ ^16^, ^17^ ^]^ However, these invasive methods may cause significant discomfort and pose risks to patients with major depressive disorder (MDD), limiting their practical application.^[^ ^18^ ^]^

Intranasal administration has emerged as a non‐invasive route for drug delivery, offering several advantages over other routes by avoiding systemic distribution and enhancing drug delivery to the brain through bypassing the BBB.^[^ ^19^ ^]^ However, the nasal epithelium and mucosal barrier pose significant challenges due to their high turnover rate and selective permeability, which can hinder the effective passage of bioactive molecules.^[^ ^20^, ^21^ ^]^ These barriers suggest that optimizing nasal‐to‐brain delivery system requires strategies that enhance the drug permeability across the nasal barrier. Studies have shown that perillyl alcohol, an anti‐cancer agent under clinical investigations, possesses the ability to overcome biological barriers by enhancing cytoplasmic membrane penetration without inducing significant cytotoxicity.^[^ ^22^ ^]^ Intranasal delivery of NEO100, a GMP‐grade version of perillyl alcohol, significantly improved the brain delivery efficiency of bortezomib and enhanced its therapeutic effect against glioblastoma multiforme (GBM) in mouse models.^[^ ^23^ ^]^ Additionally, a clinical trial demonstrated that NEO100 improved the neuropharmacokinetic of temozolomide in a patient with inoperable GBM.^[^ ^24^ ^]^ Although perillyl alcohol itself improves the delivery efficiency of hydrophobic small molecules via intranasal administration, due to its hydrophobic property, it hardly forms stable nanostructures with water‐soluble RNA molecules and facilitates the intranasal delivery of RNA therapeutics.^[^ ^25^, ^26^ ^]^ Therefore, innovative formulations are required to combine the benefits of perillyl alcohol with the delivery needs of RNA‐based drugs, ensuring both stability and efficacy.

Here, we developed novel nanoemulsion structures (PANEs) using perillyl alcohol, ionizable/cationic lipids, and surfactants to facilitate brain delivery of circDYM for the treatment of MDD. These PANEs demonstrated high RNA encapsulation efficiency and stability during long‐term storage. Specifically, the optimized formulation, PANE2‐4, exhibited significantly higher brain delivery efficiency of circDYM compared to unmodified LNP with commercialized compositions. Intranasal delivery of PANE2‐4‐circDYM successfully alleviated depressive‐like behaviors in the mouse model of LPS‐induced depression by inhibiting neuroinflammation marked by a reduction in the number of CD11b^+^CD45^dim^ microglia and iNOS expression. Treatment with PANE2‐4‐circDYM also improved neuroplasticity which was evidenced by an increase in the expression of PS‐D95 and synaptophysin in LPS‐induced mouse model of depression. This study provides an innovative approach to treat MDD using nanoemulsion‐based gene delivery and circRNA therapy, highlighting the potential of PANE2‐4 as a promising therapeutic modality for brain‐targeted treatments.

## 2 Experimental Section

### 2.1 PANE‐circDYM Formulation and Characterization

**Figure 1:**
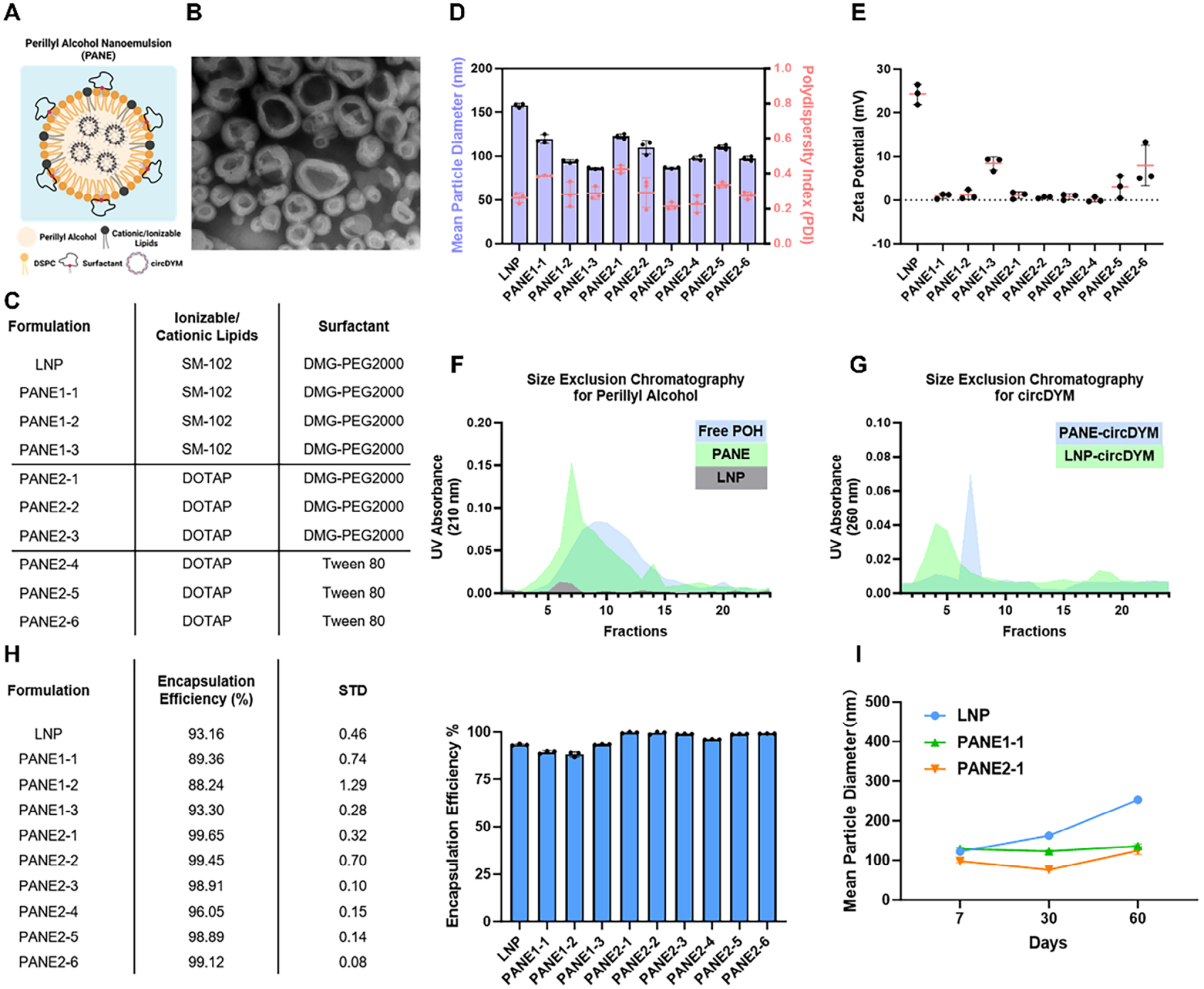
Preparation and characterization of PANEs encapsulating circDYM. A) The structure of PANEs that is composed of perillyl alcohol, cationic or ionizable lipids, DSPC, and surfactants for circDYM encapsulation delivery. B) Representative image of PANE2‐4‐circDYM. scale bar: 100 nm. C) Different compositions of PANEs in terms of cationic lipids, ionizable lipids, and surfactants. D) Mean particle diameters of LNP and PANEs encapsulating circDYM. E) Zeta potential of LNP and PANEs. Size exclusion chromatography indicating the presence of perillyl alcohol F) and circDYM encapsulation G) in PANE‐circDYM using a Sepharose CL‐4B gel column. UV absorbance at 210 nm and 260 nm were measured for the presence of perillyl alcohol and circDYM respectively. H) circDYM encapsulation efficiency (EE%) in LNP and PANEs as determined by Ribogreen RNA quantification kit and reagent. (I) Long‐term stability of LNP‐circDYM.

The IVT circDYM was synthesized by Geneseed Biotech. Co., Ltd. (Guangzhou, China). To enable visualization and tracking of circDYM, Cyanine 5 (Cy5) was chemically conjugated to circDYM, forming Cy5‐circDYM, which provides fluorescent signals for detection purposes. The Cy5‐circDYM was procured from Youhuan Biopharmaceutical Technology Co., Ltd. (Suzhou, China). DiR (CAS: 100068‐60‐8), a common fluorescent tracer used to stain nanoparticles composed of lipid layers, was purchased from Beyotime Biotechnology (Shanghai, China) to label PANEs.^[^ ^27^ ^]^ PANEs were formulated by mixing perillyl alcohol (Aladdin, China) with ionizable or cationic lipid including SM‐102 (Bidepharm, China) or 1,2‐dioleoyl‐3‐trimethylammonium propane (DOTAP) (Meryer Biochemical, China), 1,2‐distearoyl‐sn‐glycero‐3‐phosphocholine (DSPC) (Rhawn, China), surfactant including 1,2‐dimyristoyl‐rac‐glycero‐3‐methoxypolyethylene glycol‐2000 (DMG‐PEG2000) (Bidepharm, China) or Tween 80 (Kermel, China). The selection of lipids and surfactants varies across each PANE formulation, with detailed compositions provided in **Figure** **1**. The weight percentage (%) of perillyl alcohol: ionizable/cationic lipids: DSPC: surfactants were maintained at (40‐60): (10‐15): (10‐35): 10. The detailed compositions of each PANE formulation were presented in Table S1 (Supporting Information). The emulsion mixture was injected into sodium acetate buffer (50 mM) followed by probe sonication to fabricate empty PANE. Finally, empty PANE was injected into the circDYM phase in sodium acetate solution (50 mM) with a nitrogen‐to‐phosphate (N‐P) ratio of 6 to formulate PANE‐circDYM. LNP standards were formulated based on ethanol injection of SM‐102, DSPC, cholesterol (Bidepharm, China), and DMG‐PEG2000 into circDYM phase in sodium acetate buffer (25 mM). The molar percentage (%) of SM‐102: DSPC: cholesterol: DMG‐PEG2000 was maintained at 50: 10: 38.5: 1.5. The final N‐P ratio was maintained at 6.

Particle sizes and zeta potentials of PANE and LNP encapsulating circDYM were measured by dynamic light scattering (DLS) using a BeNano 180 Zeta Pro (Dandong Bettersize Instruments Ltd., China). Sepharose CL‐4B size exclusion chromatography was performed to examine the formation of PANE and circDYM encapsulation. PANE structures and circDYM were determined by UV‐Vis spectrometry at 210 nm (perillyl alcohol) and 260 nm (nucleic acid) using a NanoDrop 2000 spectrophotometer.^[^ ^28^ ^]^ The encapsulation efficiency of circDYM in PANEs and standard LNP was determined by Quant‐iT RiboGreen RNA Reagent and Kit (Thermo Fisher Scientific, MA, United States) following the manufacturer's protocols. Transmission electron microscope (TEM) images of PANE‐circDYM were obtained from Nanjing Enhancer Biotechnology Co., Ltd. (Nanjing, China).

### 2.2 Cell Culture

Mouse hippocampal neuronal (HT22) cells were grown in DMEM supplemented with 10% fetal bovine serum (FBS) and 1% penicillin/streptomycin. Human nasal epithelial (RPMI‐2650) cells were grown in MEM supplemented with 10% FBS and 1% penicillin/streptomycin. Cells were maintained at 37 °C and grown under a humidified atmosphere containing 5% CO_2_.

### 2.3 Hemolysis Assay

Mouse red blood cells (RBCs) were harvested from C57BL/6J mice and subsequently diluted 50‐fold with phosphate‐buffered saline (PBS). Mouse RBCs were further centrifuged and washed with PBS buffer twice. Then, RBCs were resuspended in PBS. PANE‐circDYM was then added to resuspended RBCs at 200 ng of circDYM per cell. The treated RBCs were incubated at 37 °C for 1 h. After incubation, RBCs were centrifuged, and the UV absorbance of hemoglobin in the supernatant was measured at 541 nm. RBCs without any treatment were designed as the negative control group, and results from all treated groups were normalized to the untreated group.

### 2.4 CircDYM Transfection by LNPs and PANEs

HT22 and RPMI‐2650 cells were seeded at 3000 cells per well in 96‐well plates or 10^5^ cells per well in 24‐well plates prior to circDYM transfection. Cells were further treated with empty PANE or PANE‐circDYM. In cellular uptake studies, HT22 and RPMI‐2650 cells were treated with PANE‐Cy5‐circDYM at 100 ng of circDYM per well for 24 h. After transfection, cells were replaced with fresh culture media, and circDYM uptake in both cell lines was determined by fluorescence intensity from Cy5‐conjugated circDYM and by DiR‐labeled LNPs and PANEs (Beyotime, China). Lipofectamine 3000 (lipo3000, Thermo Fisher, United States) was used as RNA delivery control in comparison with LNPs and PANEs. Cells without any treatment were designed as the negative control group, and cells transfected with lipo3000‐circDYM were designed as standard control group considering that both nanocarriers and circDYM were labeled with fluorescent molecules. HT22 cells were transfected with PANE‐circDYM at varied doses (50 ng, 100 ng, 200 ng of circDYM per well). After transfection, RNA samples were collected from cell lysates using TRizol reagent (Zymo Research, United States) per manufacturer protocol, and gene regulation of circDYM was determined by RT‐qPCR.

### 2.5 Cell Counting Kit‐8 (CCK8) Assay

To test the cytotoxicity of PANE‐circDYM during intranasal administration, RPMI‐2650 cells were seeded into 96‐well plates prior to treatment. Briefly, circDYM encapsulated by PANE2‐4, PANE2‐5, and PANE2‐6 were added to cells at a fixed concentration of 50 ng and 200 ng. After 72 h treatment, cell viability was determined by CCK8 (Beyotime, China).

### 2.6 Animals

Adult male C57BL/6J mice (24.0–26.0 g, 6–8 weeks old) were purchased from GemPharmatech (Nanjing, China). Before initiating experimental procedures, mice were randomly assigned to different groups and housed in 25 × 45 × 15 cm cages and maintained on a 12:12 h light/dark cycle (lights on at 07:00 AM). Food and water were available ad libitum. Mouse procedures were approved by the Institutional Animal Care and Use Committee of the Medical School, Southeast University. All mouse experiments were approved by the Institutional Animal Care and Use Committee at the Medical School of Southeast University, Nanjing, Jiangsu, China (approval ID 20 211 027 004).

### 2.7 In Vivo Live Imaging and Biodistribution Studies for Intranasal Delivery of PANE‐circDYM

DiR‐labeled PANEs and Cy5‐conjugated circDYM were used for organ‐specific localization. Mice were divided into six groups (5 mice per group) and were intranasally administered with 0.48 µg kg^−1^ of free circDYM, LNP‐circDYM, and PANE2‐4‐circDYM. At 0, 4, 12, and 24 h post‐injection, mice were anesthetized with 2% isoflurane in oxygen and imaged under IVIS Spectrum (Perkin Elmer, Beaconsfield, UK). Mice were sacrificed and their organs were further imaged ex vivo under the same instrument at 4 h post‐injection. Mice organs were further homogenized for RT‐qPCR, immunofluorescence staining, and flow cytometry analysis. To quantify the fluorescent intensity of Cy5‐circDYM, mice were perfused with 100 mL of PBS followed by 25 mL of 4% PFA at 4 h after intranasal administration of free circDYM, LNP‐circDYM, and PANE2‐4‐circDYM. Mouse brains were fixed with 4% PFA for 24 h and sliced into 30 µm thick sections. Immunofluorescence images were captured by Olympus SLIDEVIEW VS200 (Tokyo, Japan) and the fluorescent intensity was quantified by Image J software.

### 2.8 Experimental Design for Drug Treatment

Mice were intraperitoneally injected with LPS for 5 days continuously to establish an LPS‐induced depression mouse model prior to treatments. The LPS‐induced depressive mice were divided into seven groups (n = 11 per group): Control, LPS, LPS+PANE2‐4, LPS+PANE2‐4‐circDYM (16 µg kg^−1^), LPS+PANE2‐4‐circDYM (48 µg kg^−1^), LPS+PANE2‐4‐circDYM (144 µg kg^−1^), and LPS+fluoxetine (20 mg kg^−1^). Briefly, LPS (1 mg kg^−1^ day^−1^) was administered intraperitoneally once every day throughout the treatment course. PANE2‐4 control and PANE2‐4‐circDYM were intranasally administered every other day, and fluoxetine was intraperitoneally administered at 20 mg kg^−1^ every other day. Depressive‐like behavioral tests were performed on the same sets of mice. At the end of the experiment, mice were euthanized and sacrificed. Mouse brains were removed after perfusion with sterile PBS. Mouse hippocampus samples were collected and quickly stored. Peripheral blood plasma samples and internal organs (heart, liver, spleen, lungs, kidneys) were collected at −80 °C until further use.

To develop the chronic social defeat stress (CSDS) mouse model, the male CD‐1 mice (aged 4–6 months) were utilized as aggressors.^[^ ^29^ ^]^ During the 10 d CSDS procedure, the experimental mice were subjected to physical defeat with an unfamiliar resident CD1 aggressor mouse for 10 min daily. For the remainder of the day, the experimental mice were housed on the opposite side of a perforated plexiglass divider, allowing sensory interaction but preventing direct physical contact. The mice were divided into 4 groups (n = 11 per group): Control, CSDS, CSDS+PANE2‐4 and CSDS+PANE2‐4‐circDYM (48 µg kg^−1^). Briefly, the defeat session continues to be performed every day throughout treatment. PANE2‐4 and PANE2‐4‐circDYM were intranasally administered every other day.

### 2.9 Behavioral Tests

Behavioral tests were carried out in a quiet and low‐intensity environment and were scored by the same researcher. Mice were transferred to the testing room at least 3 h before behavioral tests, and the procedures were recorded by a video camera and analyzed using an ANY‐maze system (Stoelting Corporation, USA).

#### 2.9.1 Social Interaction Test (SIT)

The mice were placed into a chamber (40 cm × 40 cm × 40 cm) with an empty wire cage. During the first 2.5 min, the experimental mouse was allowed to explore the arena freely. Following this, the mouse was removed from the arena, which was then cleaned with 70% ethanol. A novel social target (CD‐1 mice for males) was placed into the wire cage, and the interaction between the experimental mouse and CD‐1 mice was recorded for another 2.5 min. A video recorder was mounted directly above the box to record video. The social interaction ratio was computed as the ratio of time the experimental mouse spent in proximity to the enclosure (designated as the social interaction zone) when the target mouse was present versus when it was absent. Mice with a social interaction ratio of ≥1 show a behavioral profile similar to control mice and were termed resilient (RES) mice, while mice with a social interaction ratio <1 were termed stress susceptible (SUS) mice.

#### 2.9.2 Sucrose Preference Test (SPT)

One bottle of 1% (w/v) sucrose solution was supplied for 3 d to habituate mice to the solution. Then, mice were exposed to both tap water and sucrose solution bottles for 24 h to obtain the sucrose preference baseline. Finally, mice were subjected to a 24‐h sucrose preference test in which tap water and sucrose solution were provided by identical bottles. The positions of the two bottles were switched every 6 h. Sucrose and water consumption were simultaneously measured. The preference to consume sucrose solution was calculated as percentage preference = [(sucrose intake/total intake) × 100]. Tests were performed by an individual blind to the animal's treatment status.

#### 2.9.3 Forced Swim Test (FST)

Mice were dropped individually into a clear cylinder (diameter: 20 cm, height: 25 cm) filled with 15 cm water maintained at 23–25 °C. After some initial, vigorous activities in the water, mice tend to acquire an immobile posture, which is characterized by motionless floating with only necessary movements to keep their heads above water. After a 2‐min habituation period, the duration of immobility was measured for 4 min. Tests were performed by an individual blind to the animal's treatment status.

#### 2.9.4 Tail Suspension Test (TST)

Mice were suspended from the ceiling of a box by adhesive tape that was placed ≈1 cm below the tip of the tail. After a 2‐min habituation period, immobility time was measured for 4 min. Tests were performed by an individual blind to the animal's treatment status.

#### 2.9.5 Open Field Test (OFT)

The OFT was conducted using an open field box with dimensions of 40 cm × 40 cm × 40 cm. The bottom of the box was divided equally into 16 identical 10 cm × 10 cm squares, where the central area was 20 cm × 20 cm and the rest were peripheral areas. A video recorder was mounted directly above the box to record video. At the beginning of the experiment, mice were placed in the center area of the box and explored freely for 4 min. The total distance the mice moved, the number of times they entered the central area, and the time they entered the center area were recorded. The box was wiped with 75% alcohol to clean it after each test. Tests were performed by an individual blind to the animals’ treatment status.

### 2.10 Reverse Transcription and Real‐time Polymerase Chain Reaction (RT‐qPCR) Assay

The plasma total RNA was reverse transcribed using the HiScript Q RT SuperMix for RT‐PCR Kit (Vazyme, R123‐01) following the manufacturer's instructions. Real‐time PCR was performed on the Applied Biosystems QuantStudio5 using the manufacturer's recommended cycling conditions with SYBR Green Real‐time PCR Master Mix (Vazyme, Q131‐01). All samples were analyzed in replicates. The circRNA transcripts were amplified using primers synthesized by Invitrogen, as listed in Table S2 (Supporting Information). The RT‐qPCR results were standardized to GAPDH control values and relative expression was calculated using the 2^−ΔΔCt^ method.

### 2.11 Flow Cytometry

Mouse brains were rapidly removed and stored in ice‐cold PBS. For cell analysis, brain tissues were digested by Papain (Worthington Biochemical, United States) in Gibico RPMI 1640 medium (Thermo Fisher, United States) at 37 °C. Cell suspension was then filtered through a 70 mm nylon mesh, and single cells were collected by centrifugation. The cells were resuspended in a 30% Percoll density gradient (GE Healthcare, China) and centrifuged for 25 min with 900 g at 4 °C temperature. Cells were obtained by collection of the bottom fraction in 30% Percoll. Cells were stained with fluorochrome‐conjugated antibodies or their corresponding isotopic controls for 30 min at 4 °C in darkness. The following antibodies were used at the manufacturer's recommended dilutions: APC anti‐mouse NCAM‐1 (FAB7820A‐100, R&D), FITC anti‐mouse/human CD11b (101 205, BioLegend), and PerCp‐Cy5.5 anti‐mouse CD45 (561 869, BD Pharmingen). The presence of PANE and circDYM can be identified by DiR and Cy5 fluorescence respectively in biodistribution studies. All samples were measured using an LSRFortessa X‐20 (BD Biosciences, United States). Data were analyzed using FlowJo version 10.8.1 (BD Biosciences, United States).

### 2.12 Biochemistry Parameters

Mice plasma samples were collected by centrifugation for 15 min at 1000 g, 4 °C. The levels of blood biochemical parameters, including aminoleucine transferase (ALT), aspartate aminotransferase (AST), urea, creatinine (Crea), creatine kinase (CK), and triglyceride (TG), were analyzed on a Chemray‐800 Auto Chemistry Analyzer according to the manufacturer's instructions (Rayto Life and Analytical Sciences Co., Ltd., China).

### 2.13 Western Blot

Lysates were harvested from cells in RIPA lysis buffer (Beyotime, China) containing a protease inhibitor cocktail. Equal amounts of protein were separated via sodium dodecyl sulfate‐polyacrylamide gel electrophoresis and were then transferred to polyvinylidene fluoride membranes electrophoretically. After incubation with blocking buffer, the membrane was incubated with antibodies against PSD‐95 (20665‐1‐AP, Proteintech group), synaptophysin (17785‐1‐AP, Proteintech group), iNOS (18985‐1‐AP, Proteintech group), and GAPDH (1:2000, 60004‐1‐lg, Proteintech group) overnight at 4 °C. After 3 washes, the membrane was incubated with a horseradish peroxidase‐conjugated goat anti‐mouse/rabbit IgG secondary antibody (1:2000, 7076P2/7074P2, Cell Signaling). Signals were detected by the Automatic Chemiluminescence Image Analysis System (Tanon 5200, Tanon Science & Technology). Quantification of the individual protein bands was performed via densitometry using Image J software.

### 2.14 Immunofluorescence Staining and Confocal Imaging

CircDYM was intranasally administered to mice in free solution or encapsulated by PANE2‐4 to investigate the intracerebral localization of circDYM. Anti‐NeuN (ab177487, Abcam), anti‐Iba‐1 (019‐19741, Wako Pure Chemicals, Japan), and anti‐GFAP (G3893, Sigma‐Aldrich, United States) antibodies were incubated separately overnight at 4 °C. After washing 3 times with PBS, the samples were incubated with Alexa Fluor 488‐conjugated Fab fragment goat anti‐mouse or rabbit IgG (H+L) (111‐547‐003, Jackson ImmunoResearch Laboratories, United States) and Alexa FluorTM 594 goat anti‐rabbit IgG (H+L) (A‐11012, Invitrogen, United States) for 1 h. After washing 3 times with PBS, the tissue sections were mounted onto glass slides with DAPI solution (0100‐20, SouthernBiotech). Images were captured by confocal microscopy (Leica TCS SP8X STED, German) and analyzed by Image J software.

### 2.15 Hematoxylin‐Eosin (HE) Staining

The major organs from mice were obtained for HE staining to assess the organ toxicities carried out by the treatment with PANE2‐4‐circDYM. Briefly, after fixation in paraformaldehyde (4%) for 48 h, the tissues were sectioned and stained with HE and finally visualized under a microscope (Olympus SLIDEVIEW VS200, Japan).

### 2.16 Statistical Analysis

Statistical analysis was performed using GrandPad Prism 10.1.2 Software. The Shapiro‐Wilk test was utilized to assess the normality of distribution within each group. For comparisons between two groups, Student's *t*‐test was applied for normally distributed data, while Mann‐Whitney *U* test was used for non‐normally distributed data. Comparisons involving multiple groups were analyzed using one‐way ANOVA followed by the Holm‐Sidak post hoc test for data with normal distribution, and the Kruskal‐Wallis test followed by Dunn's post hoc test for data with non‐normal distribution. Data collected at multiple sequential time points were analyzed using two‐way repeated‐measures ANOVA, followed by the Holm‐Sidak post hoc test. All data were presented as means±SEM. The statistical analysis used for different experiments is indicated in the figure legends, and statistical significance was set at *P* < 0.05.

### 2.17 Ethical Statement

All animals received care according to the guidelines in the Guide for the Care and Use of Laboratory Animals of Southeast University. Animal procedure was approved by the Institutional Animal Care and Use Committee (IACUC) of the Medical School, Southeast University, Nanjing, Jiangsu, China (approval ID 20211027004).

## 3 Results and Discussion

### 3.1 Development and Characterization of Different PANE Compositions Encapsulating circDYM

PANE was composed of perillyl alcohol, cationic or ionizable lipids, DSPC, and surfactants for circDYM encapsulation (Figure 1A). Unlike the single phase or onion‐like structure of the LNP‐RNA complex observed under transmission electron microscopy (TEM) or cryo‐EM, a two‐phase structure of PANE‐circDYM was observed under TEM where circDYM‐lipids complex was surrounded by other PANE components. Here, circDYM was encapsulated by the cationic lipid inside the PANEs which was recognized as the interior dark core, and other PANE components were recognized as the brighter area surrounding the circDYM (Figure 1B).^[^ ^30^, ^31^ ^]^ Our study indicated that SM‐102 and NT1‐O14B showed higher deliver efficiency of circDYM in HT‐22 cells compared with DLin‐MC3‐DMA (Figure S1, Supporting Information). Given that NT1‐O14B‐based LNP exhibited larger particle size and lower RNA encapsulation efficiency compared with other ionizable lipids according to our previous study,^[^ ^32^ ^]^ SM‐102 was chosen as our standard ionizable lipid for circDYM delivery (PANE1‐1∼1‐3) instead of NT1‐O14B and DLin‐MC3‐DMA, and DOTAP was selected as the cationic lipid (PANE2‐1∼2‐6) (Figure 1C). The surfactant among PANE formulations were selected as DMG‐PEG2000 (PANE1‐1∼1‐3 and PANE2‐1∼2‐3) or Tween 80 (PANE2‐4∼2‐6) (Figure 1C). After RNA encapsulation, SM‐102‐based PANEs exhibited smaller particle size (80‐120 nm) compared with SM‐102‐based LNPs (150‐160 nm) (Figure 1D). This is due to the absence of cholesterol and the ultrasonic nano‐emulsification of perillyl alcohol. Nevertheless, the smaller particle size of PANE‐circDYM is considered more suitable for cellular delivery compared with the larger size of LNP‐circDYM.^[^ ^33^ ^]^ The replacement of SM‐102 with DOTAP didn't change the mean particle sizes and polydispersity index of PANE significantly, suggesting that both ionizable or cationic PANE formed uniform structures with circDYM. However, reducing the amount of perillyl alcohol resulted in smaller particle sizes of PANEs (Figure 1D). This is because the presence of perillyl alcohol reduced the surface charge of nanoparticles, and therefore, decreased perillyl alcohol increased the zeta potentials of PANEs which prevent them from particle aggregations (Figure 1D,E).^[^ ^34^ ^]^

Size exclusion chromatography (SEC) indicated that perillyl alcohol was eluted along with other PANE components rather than as a free perillyl alcohol solution (Figure 1F). This again demonstrated the structure integrity during the formulation of PANEs. Both SEC and Ribogreen assays showed high circDYM encapsulation efficiency among all PANE formulations (>89%) (Figure 1G,H). Although PANE1‐1, 1–2, and 1–3 did not significantly improve the circDYM encapsulation compared with LNP using the same SM‐102 lipid, the presence of perilly alcohol enhanced the stability of PANE‐circDYM compared with LNP‐circDYM when stored at 4 °C for up to 60 days (Figure 1I). Further replacement of SM‐102 with DOTAP increased the circDYM encapsulation efficiency from 89% to 98% while maintaining equivalent long‐term stability. Therefore, DOTAP‐based PANEs (PANE2‐1∼2‐6) were preferred for circDYM encapsulation in further studies.

### 3.2 In Vitro Transfection and Cytotoxicity of PANE‐circDYM

**Figure 2:**
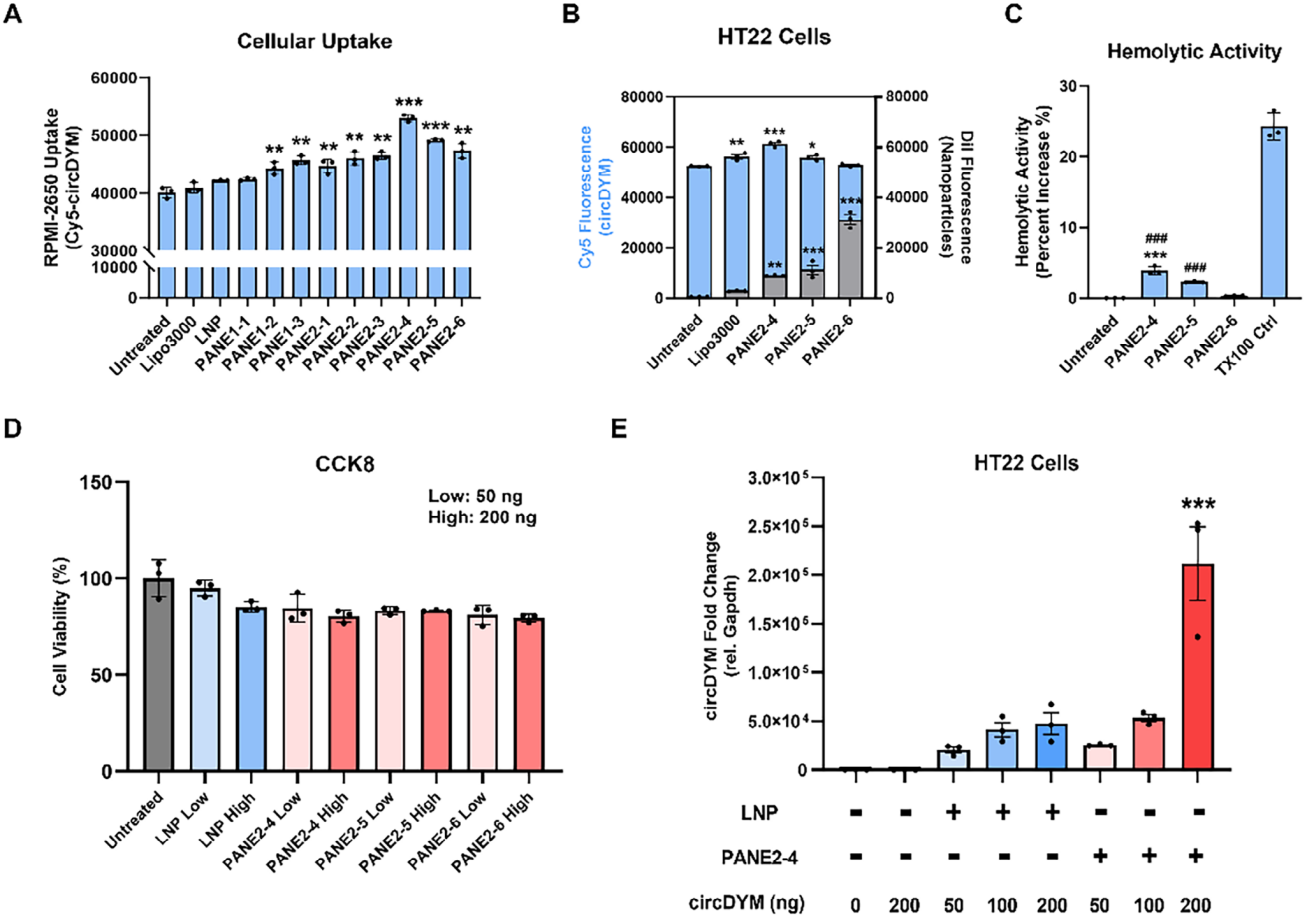
In vitro transfection efficiency and cytotoxicity of the PANE‐circDYM. A) Fluorescent intensity of Cy5‐circDYM in RPMI‐2650 cells after transfection by lipo3000, LNP, and different PANE formulations. **P < 0.01, ***P < 0.001 versus Untreated group using one‐way ANOVA followed by the Holm‐Sidak post hoc multiple comparison test. B) Fluorescent intensity of Cy5‐circDYM (blue) and DiR‐labeled PANEs (gray) in HT22 cells after transfection by lipo3000, PANE2‐4, PANE2‐5, PANE2‐6. *P < 0.05, **P < 0.01, ***P < 0.001 versus Untreated group using one‐way ANOVA followed by the Holm‐Sidak post hoc multiple comparison test. C) Hemolytic activity of PANE2‐4, PANE2‐5, and PANE2‐6 was measured in mouse RBCs to evaluate the membrane disruption ability of PANEs. The percent increase in hemolytic activity from each group was normalized to the Untreated group. ***P < 0.001 versus PANE2‐5 group; ^###^ *P* < 0.001 versus PANE2‐6 group using one‐way ANOVA followed by the Holm‐Sidak post hoc multiple comparison test. D) CCK8 assay determined the cytotoxicity of circDYM delivered by PANE2‐4, PANE2‐5, and PANE2‐6 at 50 ng and 200 ng of circDYM per well. E) The level of circDYM in HT22 cells was determined by RT‐qPCR after transfection with the different mount of PANE2‐4‐circDYM (50 ng, 100 ng, and 200 ng of circDYM per well) for 24 h. ***P < 0.001 versus free circDYM group using one‐way ANOVA followed by the Holm‐Sidak post hoc multiple comparison test. All data was presented as the mean ± SEM (n = 3).

Cellular uptake studies of Cy5‐conjugated circDYM (Cy5‐circDYM) indicated that all PANE formulations enhanced circDYM delivery to RPMI‐2650 cells compared with LNP and lipo3000 reagent which do not contain perillyl alcohol. These indicated that perillyl alcohol facilitates circDYM delivery via intranasal delivery in cell‐based studies (**Figure** **2**A). Previous research suggested that DMG‐PEG 2000 and Tween 80 are common surfactants used for RNA delivery in lipid nanoparticle and nanoemulsion respectively. Therefore, these two surfactants were chosen as the surfactant in either LNP or PANEs. To determine the optimal surfactant for circDYM delivery in PANEs, we developed both DMG‐PEG 2000‐based (PANE2‐1∼2‐3) and Tween 80‐based PANEs (PANE2‐4∼2‐6). In Figure 2A, Tween 80‐based PANEs (PANE2‐4∼2‐6) showed higher delivery efficiency of circDYM in RPMI‐2650 cells compared with DMG‐PEG2000‐based PANEs (PANE2‐1∼2‐3), suggesting that Tween 80‐based PANEs delivered more circDYM than DMG‐PEG 2000‐based PANEs in vitro. These results indicated that Tween 80 is more likely to facilitate circDYM releasement from PANE into the cytoplasmic compartment compared with PEG agents. This explains why Tween 80 was chosen as the surfactant in nanoemulsion platforms for drug delivery to brains in the previous literature.^[^ ^35^ ^]^ On the other hand, no significant difference in the brain delivery efficiency of circDYM was observed between traditional LNPs and LNPs with an extra Tween 80 component, suggesting that a combination of DMG‐PEG 2000 and Tween 80 did not significantly improve the brain delivery efficiency of circDYM in vivo (Figure S2, Supporting Information). As a potential treatment for MDD, the circDYM delivery efficiency to neurons was also evaluated in HT22 cells by 3 PANE formulations with Tween 80 (Figure 2B). These results indicated that an increased amount of perilly alcohol in PANEs increased the circDYM delivery efficiency to HT22 cells by enhancing the membrane penetration ability of PANE as determined by the hemolysis assay (Figure 2C). Here, the optimal circDYM delivery was characterized by the highest Cy5‐circDYM signals and the lowest DiR‐PANE signals after transfection of PANE‐circDYM containing the highest amount of perillyl alcohol (PANE2‐4) (Figure 2B–C). The higher membrane penetration ability of PANE induced rapid degradation of PANEs during endosomal escape and more circDYM being released into cells.^[^ ^36^ ^]^ On the other hand, the change in the amount of perillyl alcohol did not induce significant cytotoxicity of these PANEs in RPMI‐2650 cells (Figure 2D). Eventually, transfection of PANE2‐4‐circDYM successfully increased circDYM in HT22 cells, thus PANE2‐4 was selected to enhance the in vivo delivery efficiency (Figure 2E).

### 3.3 Intranasal Delivery of PANE2‐4‐circDYM to the Mouse Brain

**Figure 3:**
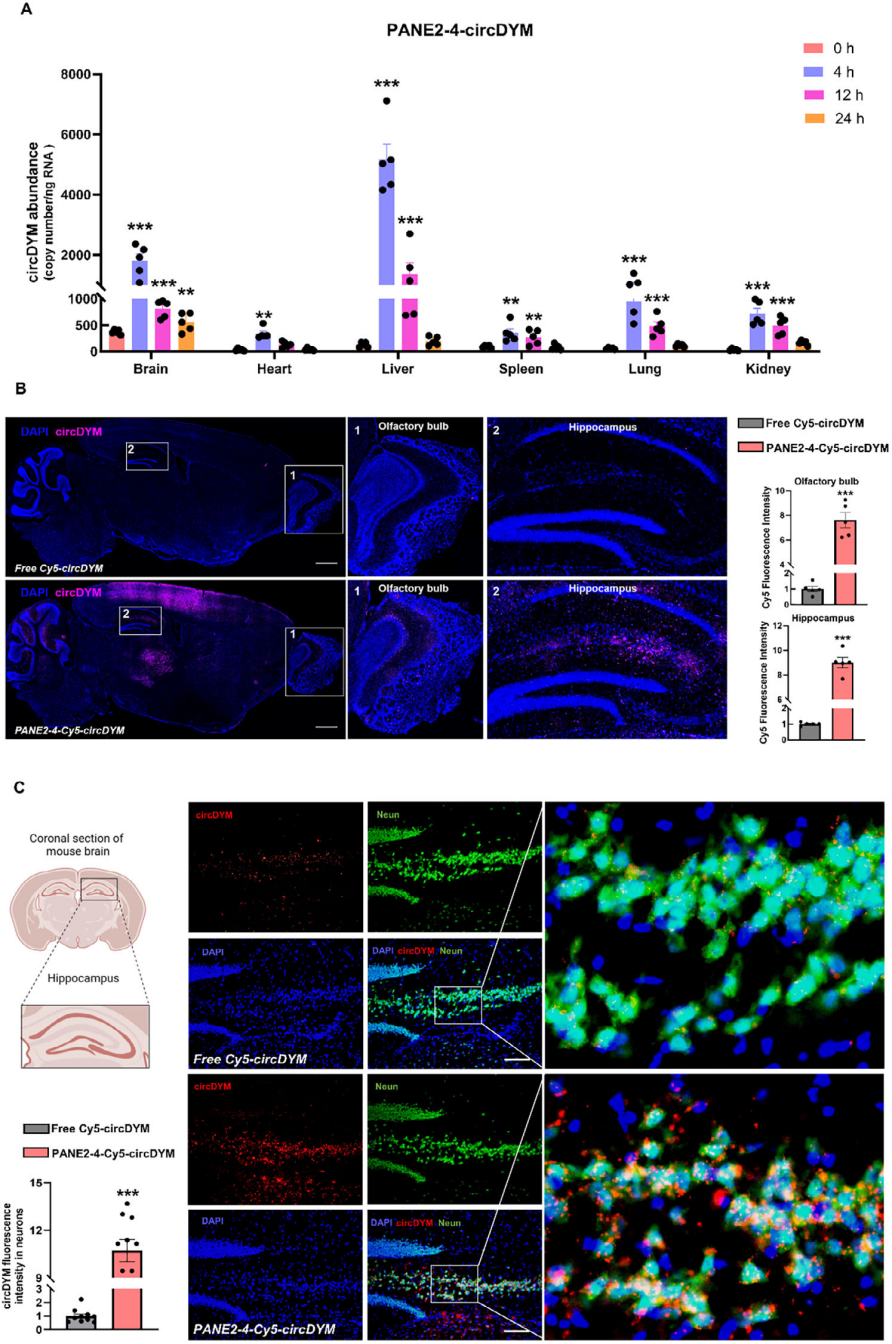
Biodistribution and hippocampal neuronal delivery of circDYM in mice after intranasal administration of PANE2‐4‐circDYM. A) Absolute qPCR assay to determine the copy number of circDYM in the brain, heart, liver, spleen, lungs, and kidneys of C57BL/6 mice at 4, 12, and 24 h after intranasal administration of PANE2‐4‐circDYM (n = 5). **P < 0.01, ***P < 0.001 versus 0 h group using one‐way ANOVA followed by the Holm‐Sidak post hoc multiple comparison test. B) Representative fluorescence images and quantification of Cy5‐circDYM fluorescence in mouse olfactory bulb and hippocampus at 4 h after intranasal administration of Free Cy5‐circDYM and PANE2‐4‐Cy5‐circDYM (n = 5). ***P < 0.001 versus free Cy5‐circDYM group using Student's *t*‐test. Scale bar: 1 mm. C) Representative images and quantifications showing colocalization of Cy5‐circDYM (red signals) and NeuN (green signals) in the hippocampal region of the mouse brain (n = 10 of randomly selected regions from 5 mice per group). Scale bar: 100 µm. ***P < 0.001 versus free Cy5‐circDYM using Student's *t*‐test. All data was presented as the mean ± SEM. h = hour.

Intranasal administration has emerged as an unconventional yet effective method for drug delivery, which avoids systemic distribution and achieved drug accumulation in the brain by bypassing the BBB.^[^ ^19^ ^]^ Consequently, numerous studies have demonstrated that this non‐invasive strategy successfully enhanced the pharmacokinetic properties of drugs within the brain.^[^ ^37^ ^]^ However, recent studies indicated that intranasal administration of lipophilic molecules and RNA therapeutics can still result in systemic distribution via the bloodstream.^[^ ^38^, ^39^ ^]^ Indeed, intranasal administration of PANE2‐4‐circDYM not only increased circDYM abundances in mouse brains but also led to elevated levels in other organs (heart, liver, spleen, lung, and kidney) at 4 h post‐treatment (**Figure** **3**A). A comparable trend was also evident from the fluorescent signals of Cy5‐circDYM and DiR‐labelled PANE2‐4 nanoparticles (Figure S3, Supporting Information). Notably, circDYM abundance peaked in all the collected organs at 24 h post‐treatment, followed by rapid systemic clearance (Figure 3A). This observation underscored the advantage of intranasal administration, which resulted in a higher accumulation of circDYM in mouse brains relative to other tissues.

Next, fluorescence imaging on a sagittal section of mice brain slices revealed that PANE2‐4 significantly increased the delivery efficiency of circDYM to mice brains compared with free circDYM (Figure 3B). Specifically, the fluorescent intensity of Cy5‐circDYM delivered by PANE2‐4 was significantly increased in mice olfactory bulbs and hippocampus compared with free Cy5‐circDYM, suggesting that PANE2‐4 successfully delivered to the hippocampus via intranasal delivery through olfactory regions (Figure 3B). Immunofluorescence imaging of the hippocampus revealed that PANE2‐4 enhanced the colocalized signals of Cy5‐circDYM and NeuN, a neuronal marker, compared to free Cy5‐circDYM (Figure 3C), which indicated successful delivery of PANE2‐4‐circDYM to neurons. Additionally, PANE2‐4‐circDYM was also detected in Iba‐1^+^ microglia, as evidenced by increased colocalization signals of Cy5‐circDYM and Iba‐1 compared to free Cy5‐circDYM (Figure S4, Supporting Information). Flow cytometric analysis further demonstrated higher Cy5‐circDYM fluorescence in CD11b^+^CD45^dim^ microglia within the brain when delivered by PANE2‐4 compared to LNP (Figure S6, Supporting Information). These results demonstrated that PANE2‐4 effectively delivered circDYM to both neurons and microglia.

### 3.4 PANE2‐4 Enhanced Brain Delivery Efficiency of circDYM Compared to LNP

**Figure 4:**
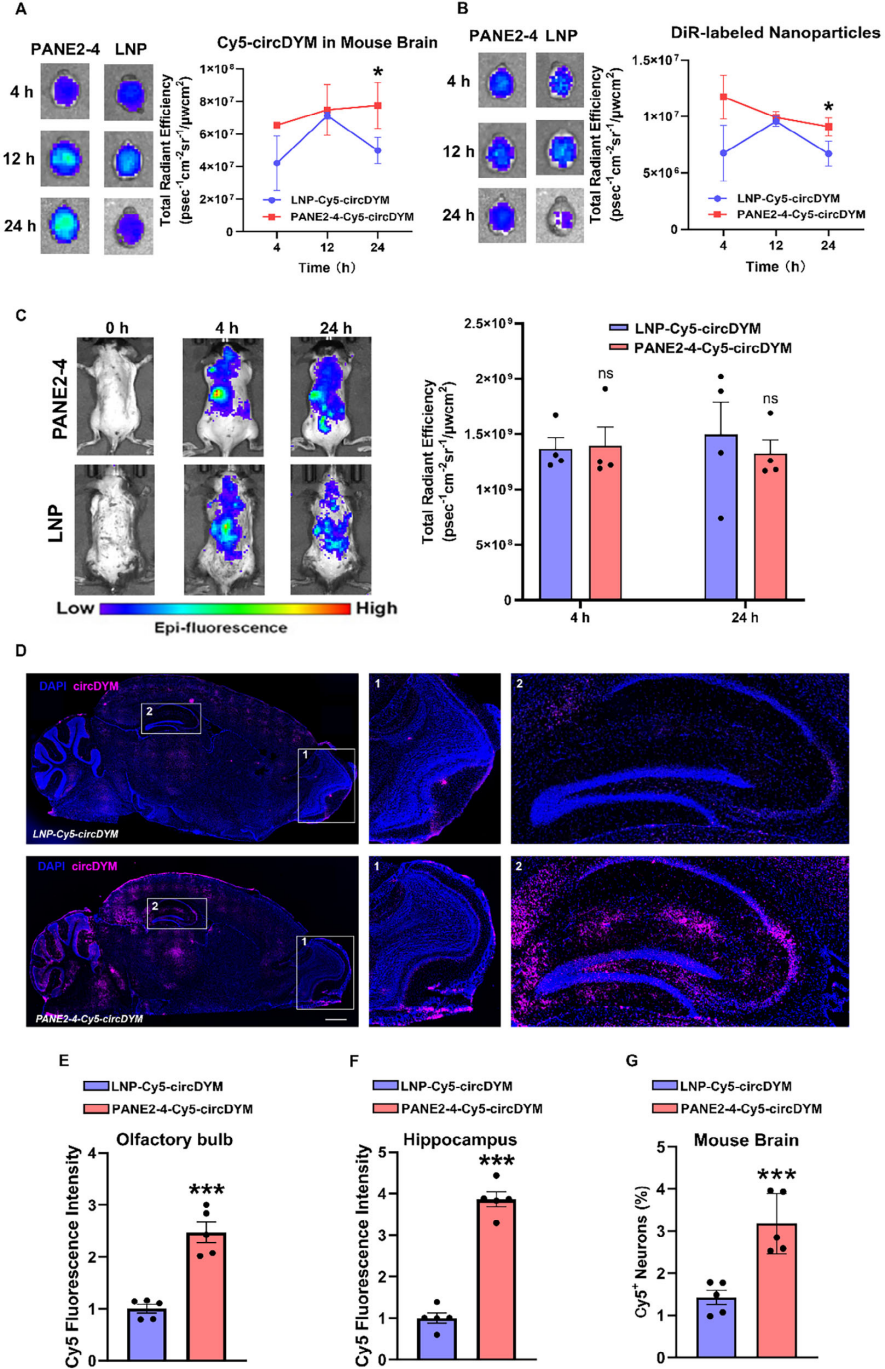
PANE2‐4 enhanced the brain delivery efficiency of circDYM compared with traditional LNP. A) Fluorescent imaging and quantification of Cy5‐circDYM in mouse brains at 4, 12, and 24 h after intranasal administration LNP‐Cy5‐circDYM and PANE2‐4‐Cy5‐circDYM (n = 5). *P < 0.05 versus LNP group using two‐way repeated‐measures ANOVA, followed by the Holm‐Sidak post hoc test. B) Fluorescent imaging and quantification of DiR‐labeled LNP and PANE2‐4 in mouse brains at 4, 12, and 24 h after intranasal administration LNP‐Cy5‐circDYM and PANE2‐4‐Cy5‐circDYM (n = 5). *P < 0.05, versus LNP group. Two‐way ANOVA followed by the Holm‐Sidak post hoc multiple comparison test was used. C) Representative in vivo fluorescence imaging and quantification of Cy5‐circDYM in mouse bodies after intranasal administration of LNP‐Cy5‐circDYM and PANE2‐4‐Cy5‐circDYM (n = 4). Data were analyzed using Mann‐Whitney *U* test. D) Representative fluorescence images and quantification of Cy5‐circDYM fluorescence in mouse olfactory bulb E) and hippocampus F) at 4 h after intranasal administration of LNP‐Cy5‐circDYM and PANE2‐4‐Cy5‐circDYM (n = 5). ***P < 0.001 versus LNP‐Cy5‐circDYM group using Student's *t*‐test. Scale bar: 1 mm. G) Flow cytometry analysis was performed to compare the fluorescent intensity of Cy5‐circDYM in mouse neurons after intranasal administration of LNP‐Cy5‐circDYM and PANE2‐4‐Cy5‐circDYM (n = 5). ***P < 0.001 versus LNP‐Cy5‐circDYM group using Student's *t*‐test. All data was presented as the mean ± SEM. h = hour.

BBB remains a major obstacle for the brain delivery of RNA therapeutics and lipid‐based nanocarriers. Unlike systemic drug administration, intranasal delivery offers an alternative route that can transport drugs directly to the brain via the olfactory and trigeminal nerves.^[^ ^19^ ^]^ However, the unmodified RNA delivery systems still face hurdles posed by the nasal epithelium and mucosal barrier. Perillyl alcohol within PANEs has been reported to facilitate drug delivery to brain by overcoming biological barriers, including both the BBB and nasal barriers.^[^ ^22^ ^]^ Several perillyl alcohol‐containing nanocarriers have been developed to enhance brain biodistribution and treat conditions such as glioma.^[^ ^26^, ^40^, ^41^ ^]^ Despite these advancements, none of these formulations were suitable to delivering RNA therapeutics and addressing neuropsychiatric diseases. In our study, after optimizing PANE formulations, we identified PANE2‐4, composed of perillyl alcohol, DOTAP, DSPC, and Tween 80, which demonstrated significantly higher brain delivery efficiency of circDYM compared with SM‐102‐based LNPs. This optimization highlights the potential of PANE2‐4 as a promising platform for the delivery of RNA therapeutics to the brain, particularly for the treatment of neuropsychiatric disorders. This was characterized by higher Cy5 and DiR signals, indicating the presence of circDYM and nanoparticles in mouse brains when delivered via PANE2‐4 compared to LNPs (**Figure** **4**A). Notably, there were no significant differences in systemic circDYM delivery between LNP and PANE2‐4 groups (Figure 4B). Therefore, the enhanced brain delivery efficiency of circDYM by PANE2‐4 can be primarily attributed to its improved penetration through nasal barriers, facilitated by the inclusion of perillyl alcohol in PANE during intranasal administration, rather than optimizations in lipid and surfactant compositions (SM‐102 versus DOTAP, DMG‐PEG2000 versus Tween 80), which may enhance systemic drug delivery (Figure 4C).^[^ ^42^ ^]^ Additionally, PANE2‐4 demonstrated significantly higher delivery efficiency of circDYM than LNPs and free circDYM in mouse brains, as evidenced by significantly higher Cy5‐circDYM fluorescent signals in the olfactory bulbs and hippocampus (Figure 4D–F). These findings further underscored the superior delivery efficiency of circDYM by PANE2‐4. Moreover, PANE2‐4 exhibited higher delivery efficiency of circDYM to neurons and microglia compared to LNP (Figure 4G; Figures S5 and S6, Supporting Information). Although the detailed molecular mechanism by which perillyl alcohol enhanced intranasal RNA therapeutic delivery requires further investigation, our results suggested that PANE2‐4 represents a more effective nanocarrier than LNPs for delivery of circDYM to treat depressive‐like behaviors in mice.

### 3.5 PANE2‐4‐circDYM Alleviated Depressive‐Like Behaviors in LPS‐Induced Mice

**Figure 5:**
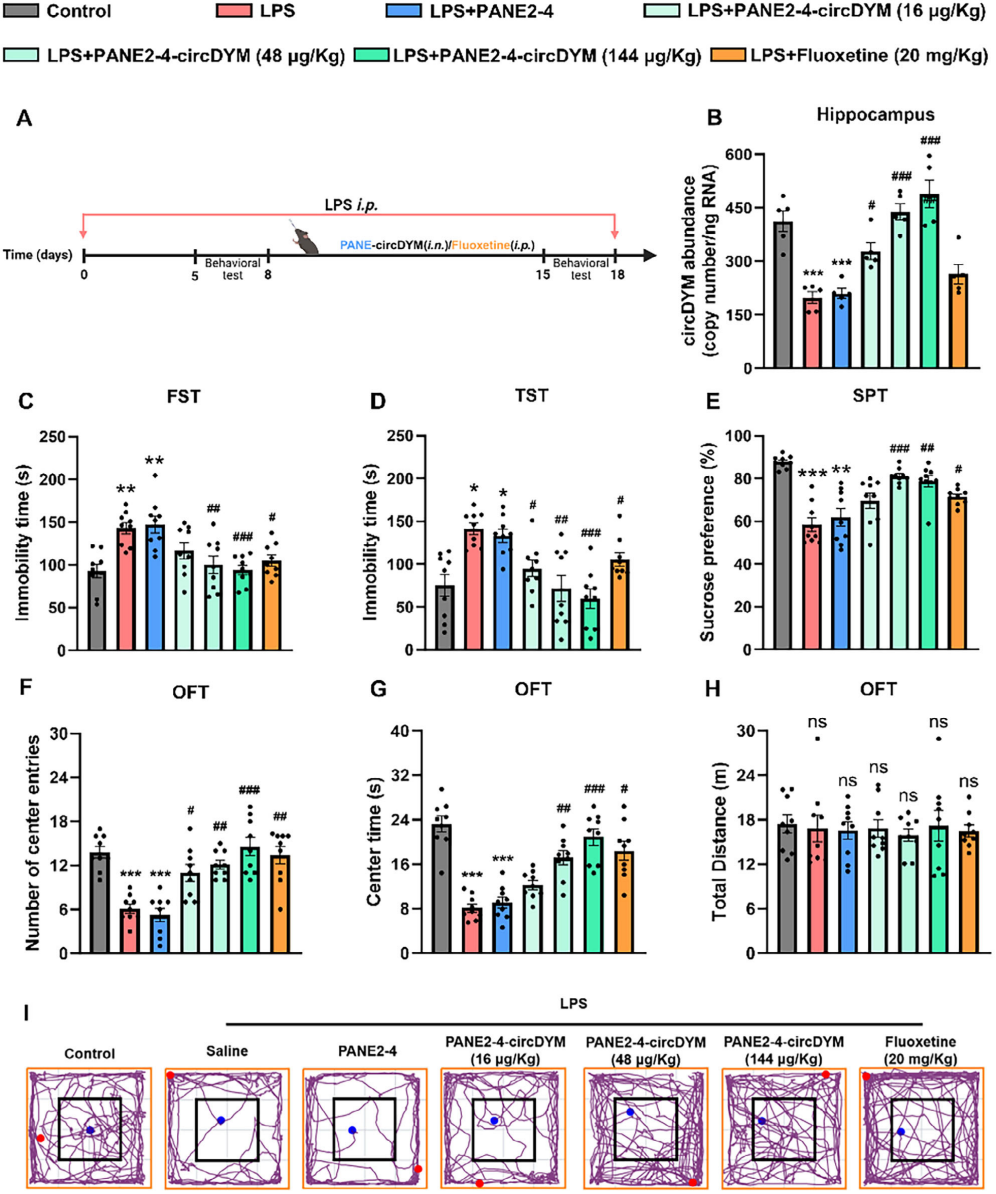
Therapeutic effects of PANE2‐4‐circDYM against depressive‐like behavior in LPS‐induced depressive‐like mice. A) Establishing LPS‐induced depressive‐like mice and treatment courses. B) circDYM abundance in hippocampus from LPS‐induced depressive mice treated with PANE2‐4‐circDYM or fluoxetine (n = 5 per group). ***P < 0.001 versus Control group; ^#^ *P* < 0.05, ^###^ *P* < 0.001 versus LPS group using one‐way ANOVA followed by the Holm‐Sidak post hoc multiple comparison test. C‐I) Intranasal administration of PANE2‐4‐circDYM ameliorated depressive‐like behaviors in LPS‐induced mice as measured by FST (C), TST (D), SPT (E), and OFT (F‐I) behavioral experiments (n = 9). *P < 0.05, **P < 0.01, ***P < 0.001 versus Control group; ^#^ *P* < 0.05, ^##^ *P* < 0.01, ^###^ *P* < 0.001 versus LPS group using one‐way ANOVA followed by the Holm‐Sidak post hoc multiple comparisons test. All data was presented as the mean ± SEM. SPT: sucrose preference test. TST: tail suspension test. FST: forced swim test. OFT: open field test.

To evaluate the therapeutic efficacy of PANE2‐4‐circDYM, LPS‐induced depression mouse model was established. After intraperitoneal injection of LPS for five consecutive days, mice exhibiting depressive‐like behaviors characterized by reduced sucrose preference and increased immobility time were selected for subsequent treatment with either PANE2‐4‐circDYM or fluoxetine (**Figure** **5**A). In this model, the level of circDYM was significantly decreased in the brains of LPS‐treated depressive mice (Figure 5B). Following treatment with PANE2‐4‐circDYM, circDYM levels were restored in a dose‐dependent manner (Figure 5B). Importantly, the increase in circDYM levels was attributed solely to PANE2‐4‐circDYM but not any component in the PANE2‐4 vehicle (Figure 5B).

Following treatment with PANE2‐4‐circDYM, depressive‐like behavior tests revealed that depressive mice exhibited decreased immobility time in FST and TST (Figure 5C,D), increased sucrose consumption in SPT (Figure 5E), and greater interest in exploring the central region of the open field (Figure 5F,G). However, no significant effect on locomotor activity was observed after PANE2‐4‐circDYM treatment (Figure 5H). Figure 5I provides the motion trajectory map of each group in the OFT. These results suggested that upregulation of circDYM by PANE2‐4‐circDYM treatment alleviated LPS‐induced depressive‐like behaviors.

The efficacy of PANE2‐4‐circDYM in ameliorating depressive‐like behaviors was also evaluated in CSDS model. This mouse model is well‐established for studying depression and has been extensively used to assess therapeutic interventions.^[^ ^43^ ^]^ C57BL/6J mice were exposed to CSDS and classified into susceptible or resilient groups based on their social interaction ratio (Figure S7A–B, Supporting Information). Susceptible mice exhibited typical depressive‐like behaviors, including prolonged immobility times in both the FST and TST, reduced sucrose consumption in the SPT, and decreased interest in exploring the central region of the open field (Figure S7C–I, Supporting Information). Treatment with PANE2‐4‐circDYM significantly reduced immobility times in both the FST and TST, restored sucrose uptake in the SPT, and increased exploratory behavior in the central region of the OFT in CSDS‐treated mice (Figure S7C–I, Supporting Information). Overall, these results indicated the potential of PANE2‐4‐circDYM as an effective therapeutic agent for depression.

### 3.6 PANE2‐4‐circDYM Ameliorated Neuroinflammation and Promoted Neuroplasticity

**Figure 6:**
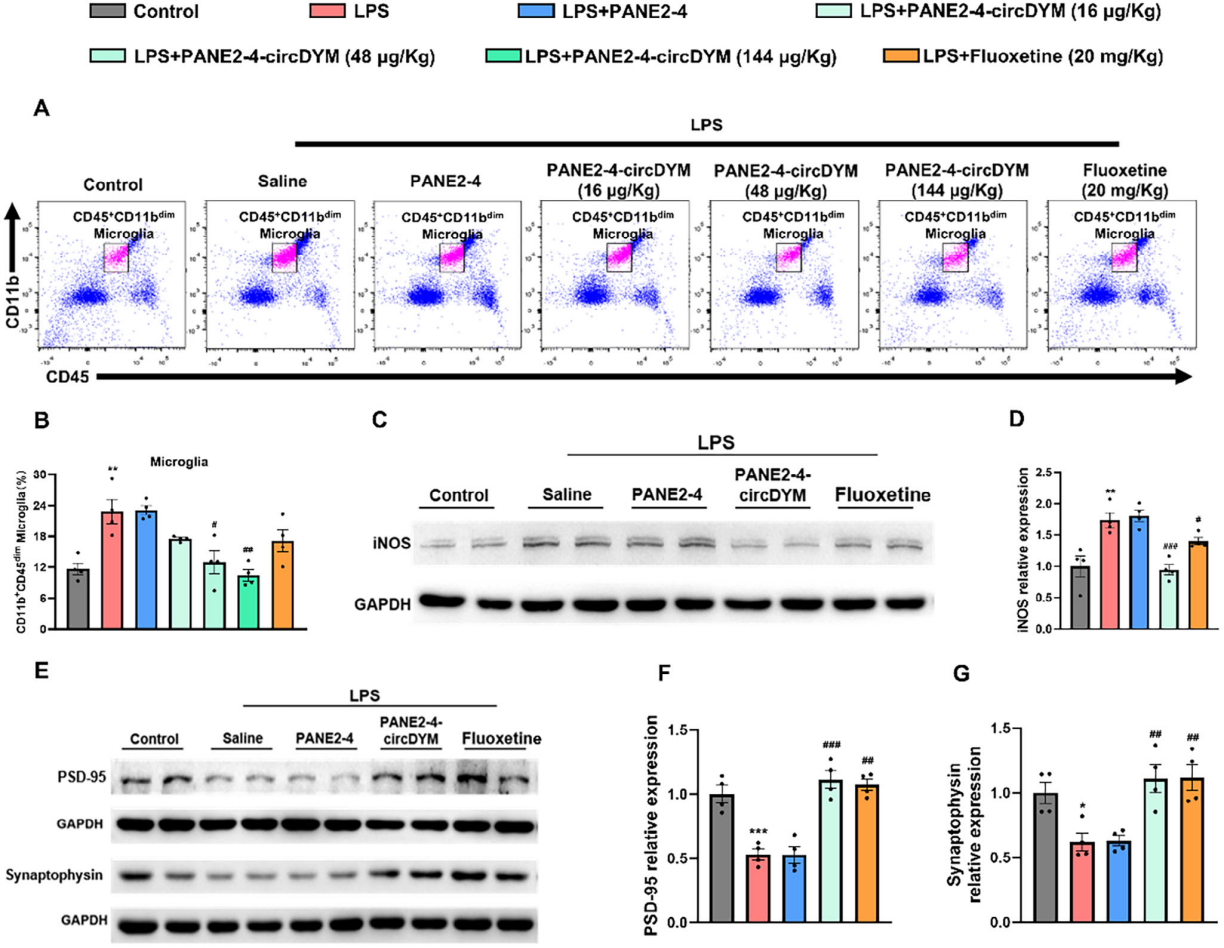
PANE2‐4‐circDYM ameliorated neuroinflammation and enhanced neuroplasticity in LPS‐induced depressive‐like mice. A) Representative flow cytograms of CD11b^+^CD45^dim^ microglia from mouse brain with different treatments. B) CD11b^+^CD45^dim^ microglia populations in mouse hippocampus (n = 4), **P < 0.01 versus Control group; ^#^ *P* < 0.05, ^##^ *P* < 0.01 versus LPS group using one‐way ANOVA followed by the Holm‐Sidak post hoc multiple comparison tests were performed. C‐D) Representative protein blots and quantifications of iNOS levels in mouse hippocampus (n = 4). **P < 0.01 versus Control group; ^#^ *P* < 0.05, ^###^ *P* < 0.001 versus LPS group using one‐way ANOVA followed by the Holm‐Sidak post‐hoc multiple comparison test. E‐G) Representative protein blots and quantifications of PSD‐95 and Synaptophysin levels in mouse hippocampus (n = 4). *P < 0.05, ***P < 0.001 versus Control group; ^##^ *P* < 0.01, ^###^ *P* < 0.001 versus LPS group using one‐way ANOVA followed by the Holm‐Sidak post‐hoc multiple comparison test. All data are presented as the mean ± SEM.

One of the hallmark features of MDD is microglia‐mediated neuroinflammation, characterized by an increased number of activated microglia and elevated expression of inducible nitric oxide synthase (iNOS).^[^ ^44^ ^]^ LPS‐induced depressive mice exhibited microglia activation, as evidenced by the increased number of CD11b^+^CD45^dim^ microglia (**Figure** **6**A–B; Figure S5, Supporting Information) and elevated iNOS expression in the mouse hippocampus (Figure 6C–D). Treatment with PANE2‐4‐circDYM significantly reduced both the number of CD11b^+^CD45^dim^ microglia and iNOS expression, indicating that PANE2‐4‐circDYM alleviated depressive‐like behaviors by suppressing neuroinflammations. Meanwhile, impaired neuroplasticity, alongside microglia‐mediated neuroinflammation, plays a critical role in the pathophysiology of depression, manifesting as synaptic loss in the hippocampus.^[^ ^45^ ^]^ Postsynaptic density PSD‐95 and synaptophysin are key synaptic proteins that regulate neurotransmitter receptors, adhesion molecules, ion channels, and signaling molecules at postsynaptic and presynaptic sites, respectively.^[^ ^46^, ^47^ ^]^ Previous studies have shown that depression is associated with reduced expression of PSD‐95 and synaptophysin, indicative of synaptic loss.^[^ ^48^ ^]^ Expression of PSD‐95 and synaptophysin was reduced in the hippocampus of LPS‐induced depressed mice compared with the control group. Treatment with PANE2‐4‐circDYM significantly restored the expressions of PSD‐95 and synaptophysin (Figure 6E–G). These findings suggested that PANE2‐4‐circDYM alleviated depressive‐like behaviors not only by mitigating neuroinflammation but also by reducing synaptic loss.

### 3.7 Safety Evaluation of PANE2‐4‐circDYM

**Figure 7:**
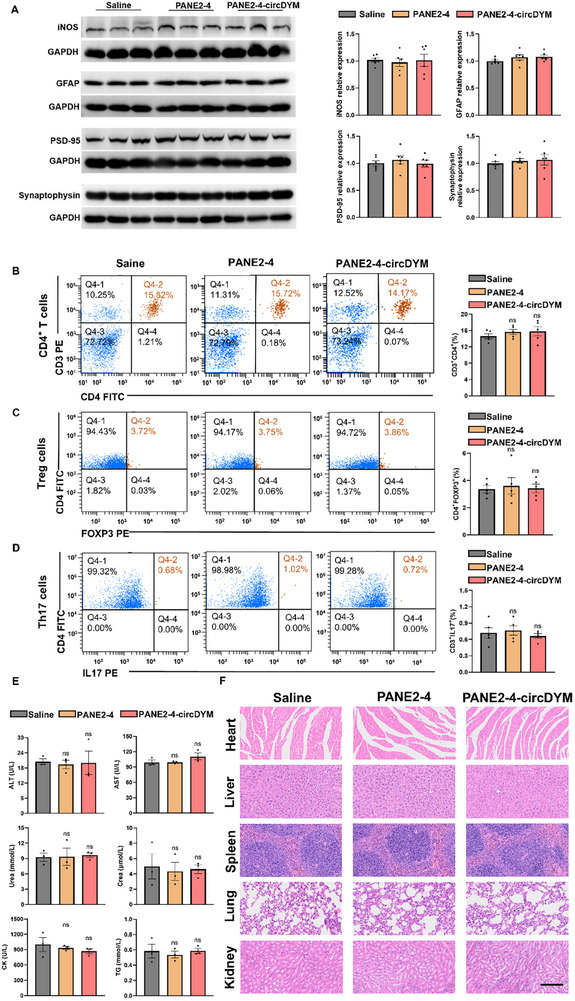
The PANE platform does not induce toxicity on the immune or nervous systems. A) Representative protein blots and quantifications of GFAP, iNOS, PSD‐95, and Synaptophysin levels in mouse hippocampus after treatment with saline, PANE2‐4, or PANE2‐4‐circDYM (n = 6). Data were analyzed using one‐way ANOVA followed by the Holm‐Sidak post hoc multiple comparison test. B–D) Peripheral immune cells were analyzed by flow cytometry indicating no overt changes in CD4^+^ T cells (B), immunosuppressive Treg cells (C), and pro‐inflammatory Th17 cells (D) in mice treated with PANE2‐5 or PANE2‐5‐circDYM versus those treated with saline (n = 6). Data were analyzed using one‐way ANOVA followed by the Holm‐Sidak post hoc multiple comparison test. E) Plasma levels of ALT, AST, Urea, Crea, CK, and TG from mice treated with saline, PANE2‐4, or PANE2‐4‐circDYM were measured by auto chemical analyzer (n = 3). Data were analyzed using one‐way ANOVA followed by the Holm‐Sidak post hoc multiple comparison test. F) H&E staining images of the heart, liver, spleen, lungs, and kidneys of mice treated with saline, PANE2‐4, or PANE2‐4‐circDYM. Scale bar: 200 µm. All data are presented as the mean ± SEM. ALT: Aminoleucine transferase. AST: Aspartate aminotransferase. Crea: Creatinine. CK: Creatine kinase. TG: Triglyceride.

Despite the nanoemulsion system being recognized as an attractive delivery platform for bioactive molecules, systemic and organ toxicities have impeded its clinical translation, particularly when the components contain cationic lipids for the therapeutic delivery of nucleic acid payloads.^[^ ^49^ ^]^ Given that PANE2‐4‐circDYM was distributed throughout all mouse organs even via intranasal administration, the blood biochemical parameters were measured in mouse serum to evaluate potential systemic toxicity. To investigate the potential toxicity or other side effects associated with the long‐term use of PANE2‐4‐circDYM, we conducted comprehensive safety assessments in mice treated with PANE2‐4‐circDYM for 7 days. First, we examined the expression of astrocyte marker GFAP, neuroinflammatory marker iNOS, and neuroplasticity proteins PSD‐95 and synaptophysin in the hippocampus of mice using Western blot analysis (**Figure** **7**A). These results suggested that PANE did not exert significant effect on the iNOS, GFAP, PSD‐95 or synaptophysin expression. Subsequently, we measured the changes in the population of CD4^+^ T cells, Treg cells, and TH17 cells in the peripheral blood of mice treated with saline, PANE2‐4, and PANE2‐4‐circDYM using flow cytometry (Figure 7B–D). Our results indicated that neither PANE nor PANE‐circDYM caused adverse effects on the immune system. No significant changes were observed in key biochemical parameters (ALT, AST, Urea, Crea, CK, and TG) between mice treated with PANE2‐4‐circDYM treated and control group (Figure 7E). Finally, to evaluate the impact of prolonged administration of PANE on peripheral organs, we performed hematoxylin and eosin (H&E) staining on the tissues (heart, liver, spleen, lung, and kidney) of mice treated with saline, PANE2‐4, and PANE2‐4‐circDYM. No significant organ toxicities were observed in these mice (Figure 7F). These findings collectively provide evidence that PANE2‐4‐circDYM does not induce significant toxicities in either the immune or nervous systems at the dosages used in this study. This supports the safety profile of the PANE platform for long‐term therapeutic applications, particularly in the context of neurological conditions, suggesting its potential suitability for therapeutic applications without inducing adverse effects on critical physiological systems.

## 4 Conclusion

MDD severely impairs patients' social and psychological performance. However, the lack of an efficient brain delivery system for RNA therapeutics limits the potential of these treatments. In this study, we developed PANEs for the therapeutic delivery of circDYM to the mouse brain. The PANE formulations effectively utilized cationic and ionizable lipids to achieve high RNA encapsulation and delivery efficiency. Notably, optimized PANE2‐4 demonstrated significantly higher circDYM delivery efficiency compared with traditional LNP, attributed to the presence of perillyl alcohol. Although the detailed molecular mechanism by which perillyl alcohol enhanced intranasal delivery of PANEs require further investigation, our findings indicated that PANE2‐4‐circDYM successfully enhanced circDYM delivery to the mouse brain and alleviated depressive‐like behaviors in the LPS‐induced depressive mouse model. Importantly, histological assessments revealed no significant organ toxicities, supporting the safety profile of PANE2‐4‐circDYM. The current study proposed PANEs as a safe and effective RNA delivery system, highlighting the therapeutic potential of PANE2‐4‐circDYM against MDD. This work not only addresses the critical need for efficient delivery systems for RNA therapeutics but also opens new avenues for treating psychiatric disorders through targeted delivery to the central nervous system.

## Conflict of Interest

The authors declare no conflict of interest.

## Author Contributions

F.G. and Z.Z. contributed equally to this work. Conceptualization: H.Y. and Z.Z.; Methodology: Z.Z. and F.G.; Software: Z.Z. F.G. and H.Y.; Validation: F.G., M.J., L.B., H.W., N.C, S.Z., and Y.W.; Formal analysis; Z.Z. and F.G.; Writing: Z.Z., F.G., Y.Z, and H.Y.; Supervision: H.Y., Y.Z., Y.J., and L.S.; Funding acquisition; H.Y. Y.Z., and Z.Z. All authors have read and agreed to the published version of the manuscript.

## Supporting information



Supporting Information

## Data Availability

The datasets used and/or analyzed during the current study are available from the corresponding author on reasonable request.
