# Supplementary material for: Therapeutic Delivery of circDYM by Perillyl Alcohol Nanoemulsion Alleviates LPS‐Induced Depressive‐Like Behaviors
Source: Adv Sci (Weinh). 2025 Mar 27;12(21):2414559. doi: 10.1002/advs.202414559 (PMC12140362; doi:10.1002/advs.202414559)
Supplement: Supplementary file 1 — Supporting Information [file ADVS-12-2414559-s001.docx]

**Therapeutic Delivery of circDYM by Perillyl Alcohol Nanoemulsion Alleviates LPS-induced Depressive-like Behaviors**

Feng Gao^a†^, Zhongkun Zhang^a†*^, Minzi Ju^a^, Liang Bian^a^, Huijuan Wang^a^, Sibo Zhao^a^, Ningbo Cai^a^, Yu Wang^a^, Yanpeng Jia^a^, Ling Shen^a^, Yuan Zhang^a^**^*^**, and Honghong Yao^a,b,c^**^*^**

^a^Department of Pharmacology, Jiangsu Provincial Key Laboratory of Critical Care Medicine, School of Medicine, Southeast University, Nanjing, Jiangsu, 210009, China.

^b^Co-innovation Center of Neuroregeneration, Nantong University, Nantong, Jiangsu, 226001, China.

^c^Institute of Life Sciences, Key Laboratory of Developmental Genes and Human Disease, Southeast University, Nanjing, Jiangsu, 210096, China.

**†These authors contributed equally to this work.**

***To whom correspondence should be addressed:**

Zhongkun Zhang, Ph.D., Department of Pharmacology, Jiangsu Provincial Key Laboratory of Critical Care Medicine, School of Medicine, Southeast University, Nanjing, 210009, Jiangsu, China. E-mail: zhongkunzhang@seu.edu.cn

Yuan Zhang, Ph.D., Department of Pharmacology, Jiangsu Provincial Key Laboratory of Critical Care Medicine, School of Medicine, Southeast University, Nanjing, 210009, Jiangsu, China. E-mail: yuanzhang@seu.edu.cn

Honghong Yao, Ph.D., Department of Pharmacology, Jiangsu Provincial Key Laboratory of Critical Care Medicine, School of Medicine, Southeast University, Nanjing, 210009, Jiangsu, China. E-mail: yaohh@seu.edu.cn

**Supplement Table S1.** Weight percentages of each component in PANE formulations.

| Weight Percentage (%) | Perillyl Alcohol | Ionizable/Cationic Lipids | DSPC | Surfactants |
| --- | --- | --- | --- | --- |
| PANE1-1 | 0.60 | 0.15 | 0.15 | 0.10 |
| PANE1-2 | 0.53 | 0.14 | 0.22 | 0.10 |
| PANE1-3 | 0.40 | 0.12 | 0.37 | 0.10 |
| PANE2-1 | 0.60 | 0.15 | 0.15 | 0.10 |
| PANE2-2 | 0.53 | 0.14 | 0.22 | 0.10 |
| PANE2-3 | 0.40 | 0.12 | 0.37 | 0.10 |
| PANE2-4 | 0.60 | 0.15 | 0.15 | 0.10 |
| PANE2-5 | 0.53 | 0.14 | 0.22 | 0.10 |
| PANE2-6 | 0.40 | 0.12 | 0.37 | 0.10 |

**Supplement Table S2. Primers sequences.**

|  | **Primers** | **Primer sequence (5’ – 3’)** |
| --- | --- | --- |
| circDYM | circDYM (Mouse)-F  circDYM (Mouse)-R | GAAGAAAAGTCCCCCGGCAG  AAGACCTTAGTTAGCGCAGCA |
| GAPDH | GAPDH (Mouse)-F | AGGTCGGTGTGAACGGATTTG |
|  | GAPDH (Mouse)-R | TGTAGACCATGTAGTTGAGGTCA |


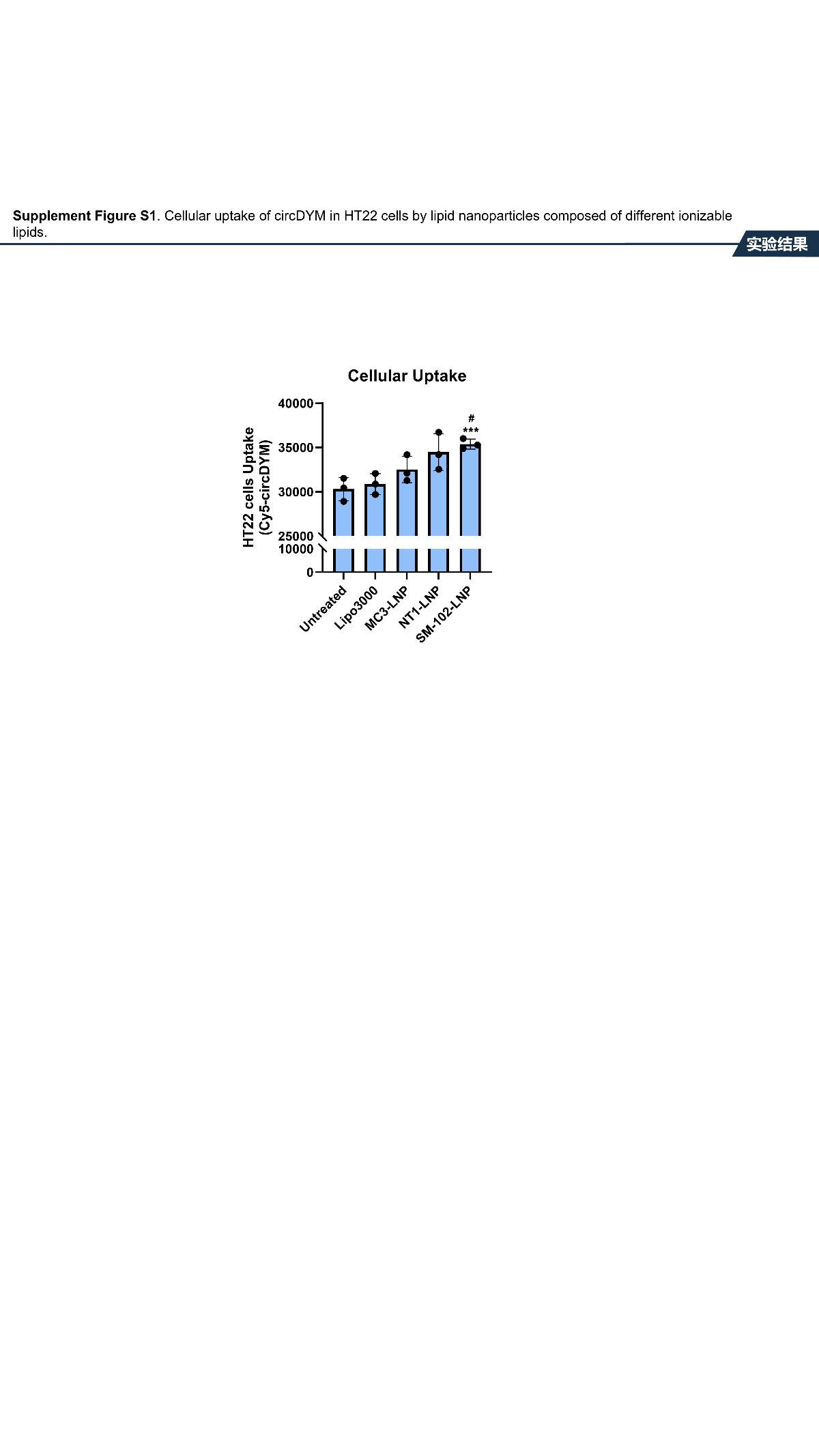


**Supplement Figure S1. Cellular uptake of circDYM in HT22 cells by lipid nanoparticles composed of different ionizable lipids.** Fluorescent intensity of Cy5-circDYM were quantified in HT22 cells after transfection by lipo3000, MC3-LNP, NT1-LNP, and SM-102-LNP (n = 3). *** *P* < 0.001 versus untreated group; ^#^ *P* < 0.05 versus MC3-LNP group using one-way ANOVA followed by the Holm-Sidak post hoc multiple comparisons test. All data are presented as the mean ± SEM.


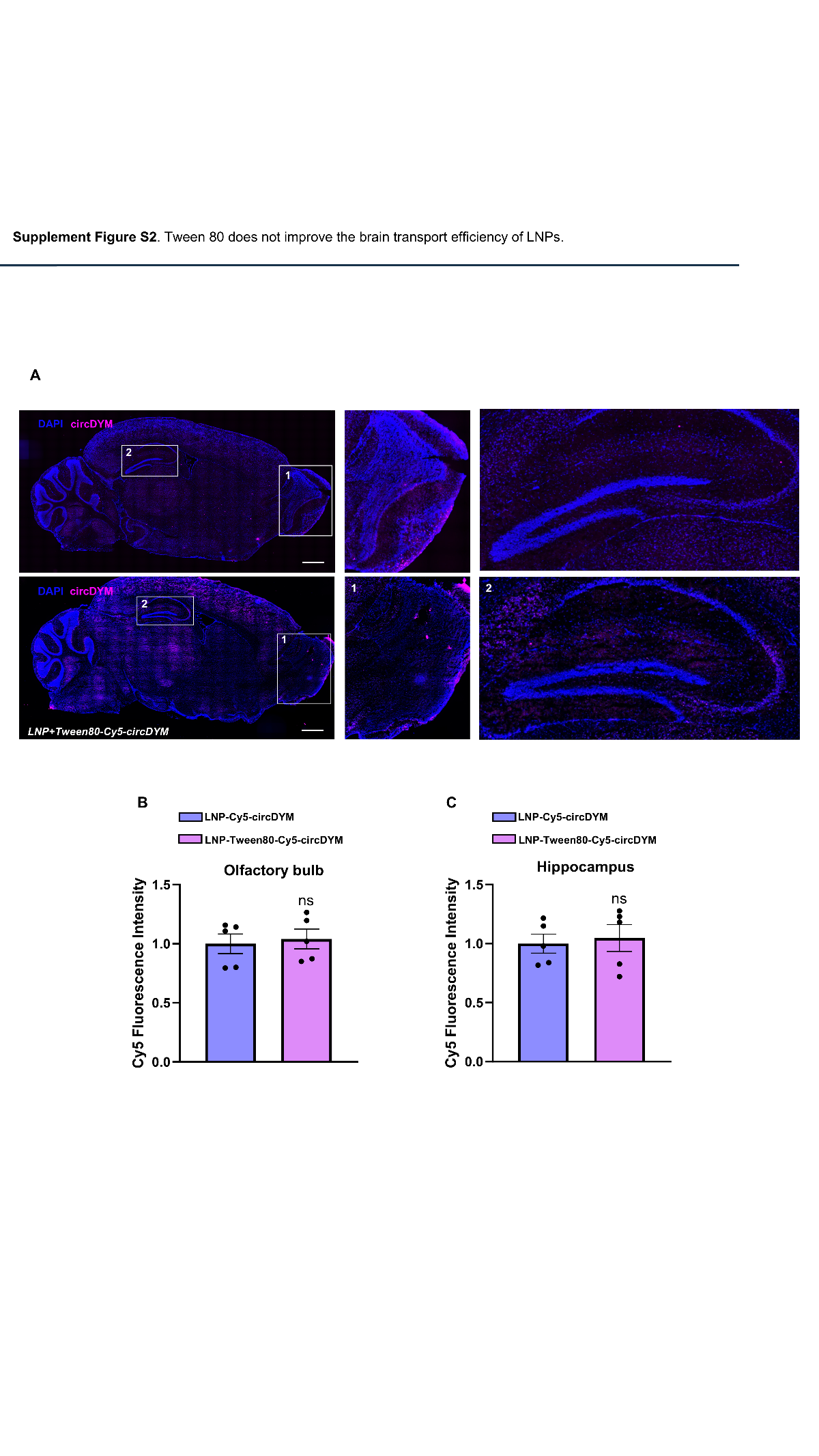


**Supplement Figure S2. Brain delivery efficiency of circDYM.** (A) Representative fluorescence images and (B, C) quantification of Cy5-circDYM fluorescence in mouse olfactory bulb and hippocampus at 4 h after intranasal administration of LNP-Cy5-circDYM and LNP+Tween80-Cy5-circDYM. Data were analyzed using Student's *t*-test. Scale bar: 1 mm. All data are presented as the mean ± SEM (n = 5).

**
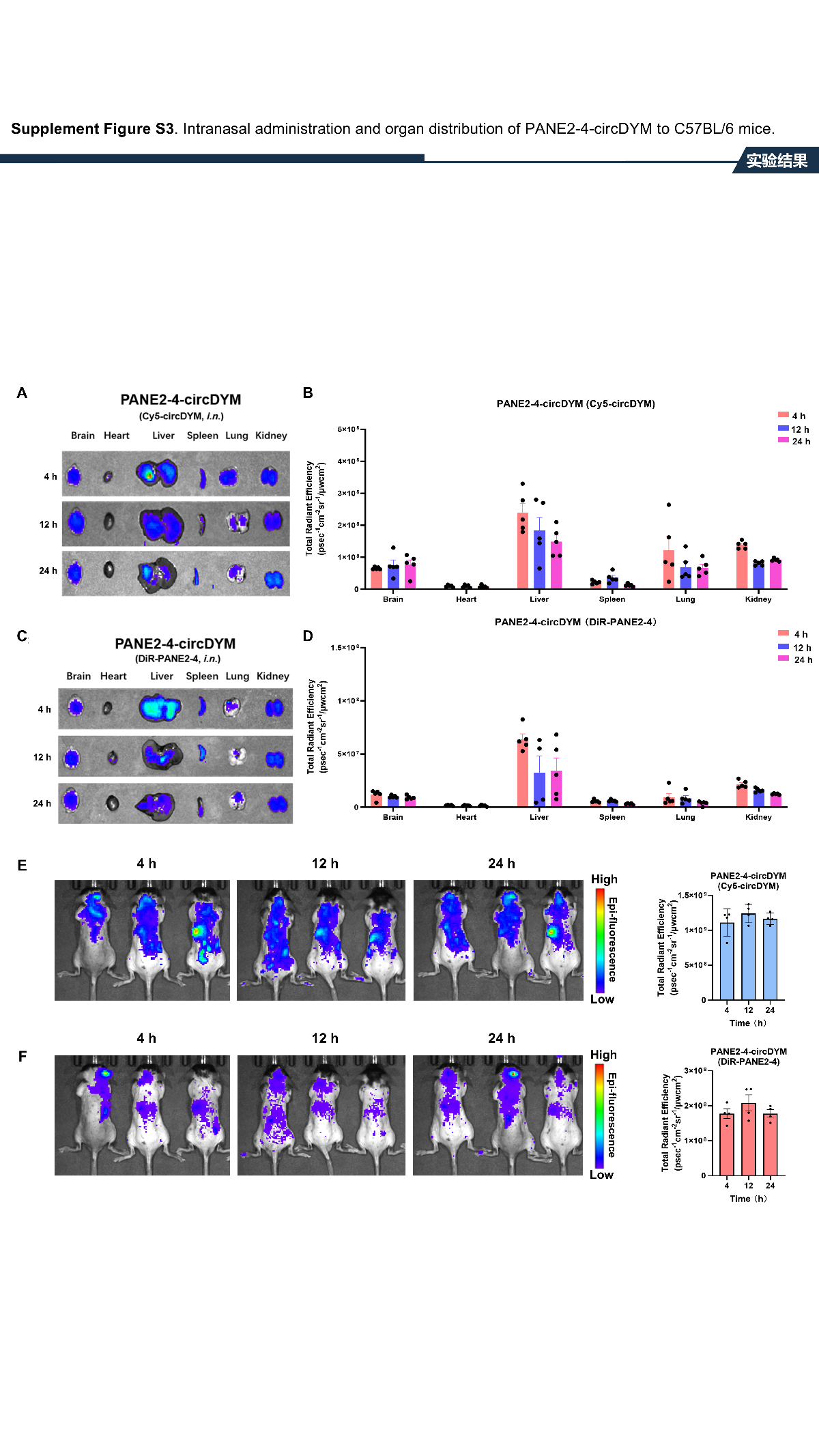
**

**Supplement Figure S3. Intranasal administration and organ distribution of PANE2-4-circDYM to C57BL/6 mice.** (A) Representative fluorescence images and (B) quantification of Cy5-circDYM fluorescence in mouse brains, hearts, livers, spleens, lungs, and kidneys at 4, 12, and 24 h after intranasal administration of PANE2-4-Cy5-circDYM (n = 5). (C) Representative fluorescence images and (D) quantification of DiR-labeled PANE2-4 fluorescence in mouse brains, hearts, livers, spleens, lungs, and kidneys at 4, 12, and 24 h after intranasal administration of PANE2-4-Cy5-circDYM (n = 5). (E) *In vivo* fluorescence imaging and quantification of Cy5-circDYM at 4, 12, and 24 h after intranasal administration of PANE2-4-Cy5-circDYM (n = 4). (F) *In vivo* fluorescence imaging and quantification of DiR-labeled PANE2-4 at 4, 12, and 24 h after intranasal administration of PANE2-4-Cy5-circDYM (n = 4). Data were analyzed using Kruskal-Wallis test followed by Dunn’s post hoc test. All data are presented as the mean ± SEM. h = hour.


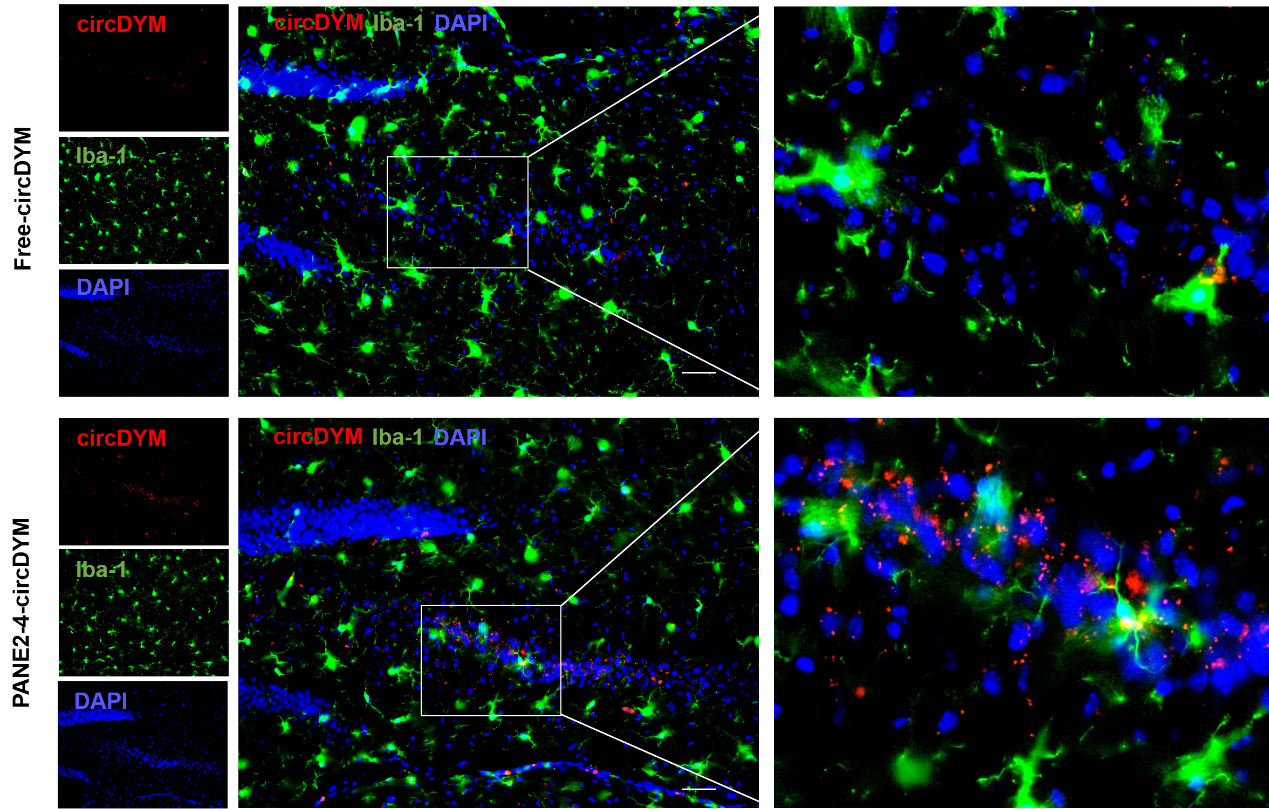


**Supplement Figure S4. Immunofluorescence colocalization of Cy5-circDYM and Iba-1 in the hippocampal region from mouse brains.** Red fluorescent signals represent Cy5-circDYM, and green fluorescent signals represent Iba-1. Scale bar: 50 μm.


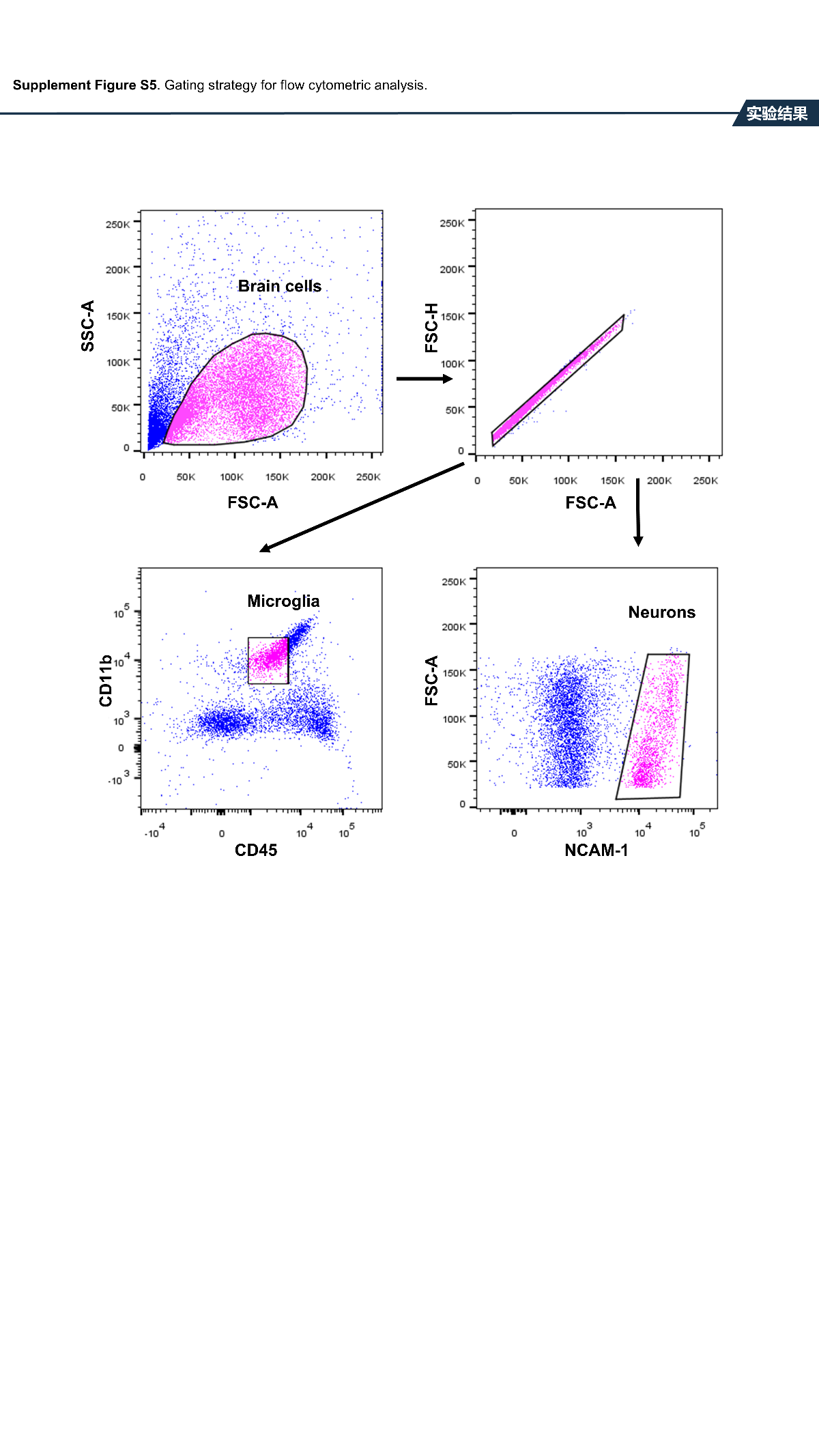


**Supplement Figure S5. Gating strategy for flow cytometric analysis.** Gating strategy for flow cytometric analysis of microglia and neurons in the brain.


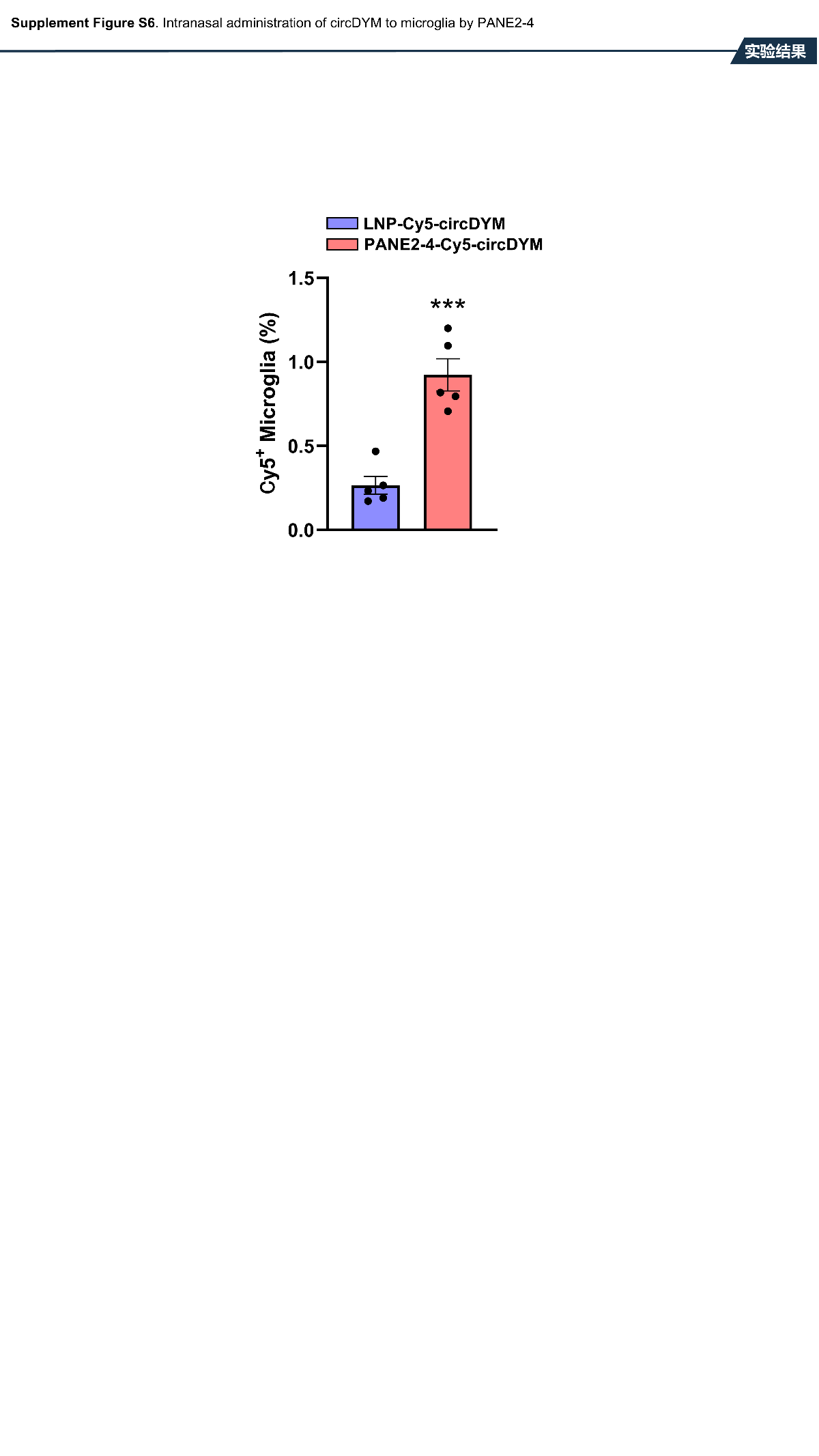


**Supplement Figure S6. Intranasal administration of circDYM to microglia by PANE2-4.** Flow cytometry analysis to compare the amount of Cy5-circDYM delivered to microglia by LNP and PANE2-4 (n = 5). *** *P* < 0.001 versus the LNP group using Student's *t*-test. All data are presented as the mean ± SEM.


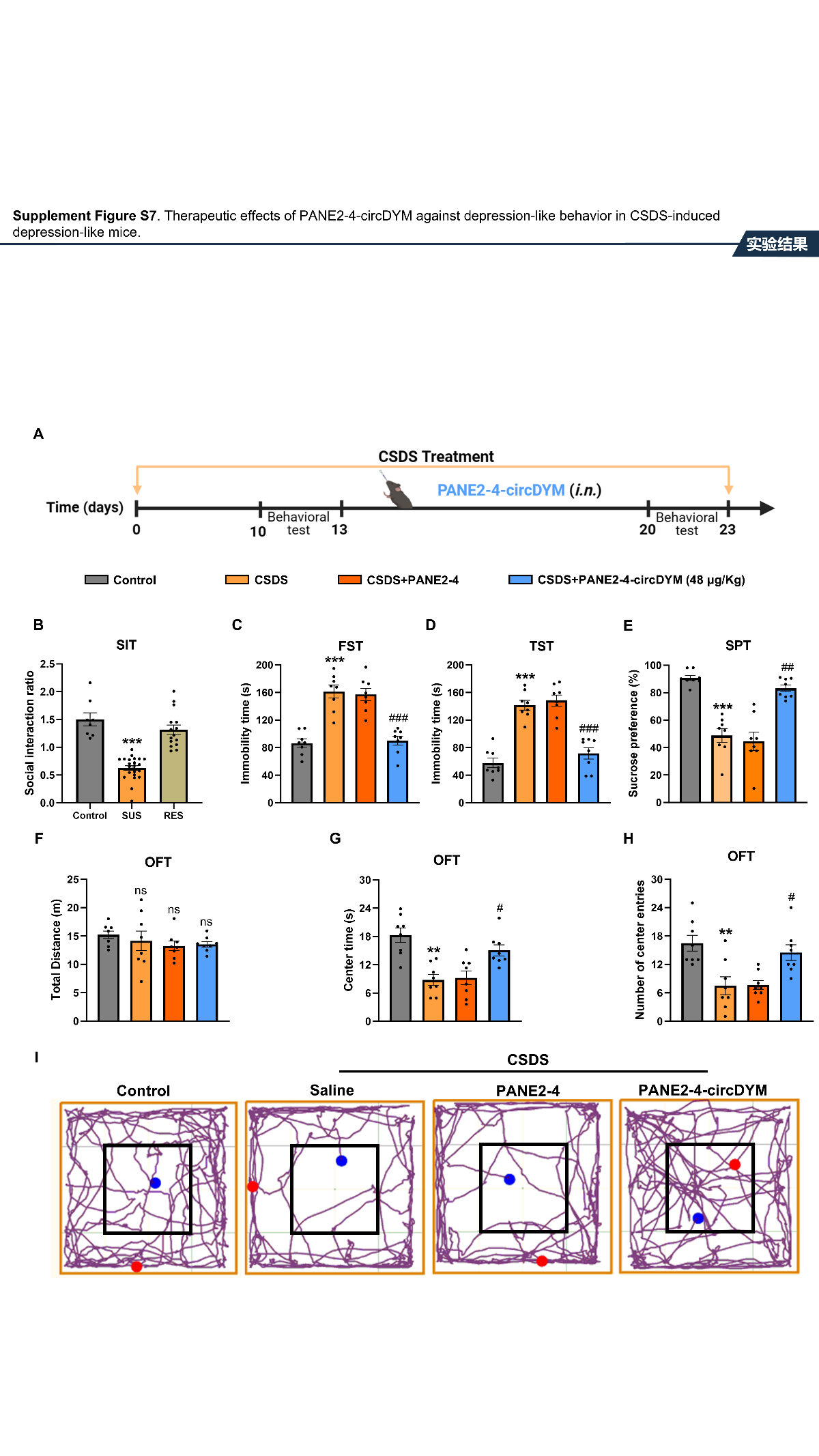


**Supplement Figure S7. Therapeutic effects of PANE2-4-circDYM against depression-like behavior in CSDS-induced depression-like mice.** (A) Establishing CSDS-induced depressive-like mice and treatment courses. (B) SIT for screening SUS and RES mice. (C-I) Intranasal administration of PANE2-4-circDYM ameliorated depressive-like behaviors in CSDS mice as measured by FST (C), TST (D), SPT (E), and OFT (F-I) behavioral experiments (n = 8). ** *P* < 0.01, *** *P* < 0.001 versus Control group; ^#^ *P* < 0.05, ^##^ *P* < 0.01, ^###^ *P* < 0.001 versus CSDS group using one-way ANOVA followed by the Holm-Sidak post hoc multiple comparisons test. All data are presented as the mean ± SEM. SIT: social interaction test. RES: resilient. SUS: susceptible. SPT: sucrose preference test. TST: tail suspension test. FST: forced swim test. OFT: open field test.
